# Uptake of alpha‐synuclein preformed fibrils is suppressed by inflammation and induces an aberrant phenotype in human microglia

**DOI:** 10.1002/glia.24626

**Published:** 2024-10-22

**Authors:** Jonna Niskanen, Sanni Peltonen, Sohvi Ohtonen, Mohammad Feroze Fazaludeen, Kelvin C. Luk, Luca Giudice, Jari Koistinaho, Tarja Malm, Gundars Goldsteins, Katrina Albert, Šárka Lehtonen

**Affiliations:** ^1^ A.I. Virtanen Institute for Molecular Sciences University of Eastern Finland Kuopio Finland; ^2^ Center for Neurodegenerative Disease Research, Department of Pathology and Laboratory Medicine University of Pennsylvania, Perelman School of Medicine Philadelphia PA USA; ^3^ Helsinki Institute of Life Science University of Helsinki Helsinki Finland; ^4^ Drug Research Program, Division of Pharmacology and Pharmacotherapy University of Helsinki Helsinki Finland; ^5^ Neuroscience Center University of Helsinki Helsinki Finland

**Keywords:** alpha‐synuclein preformed fibrils, autophagy, hiPSCs, microglia, Parkinson's disease, phagocytosis

## Abstract

Microglia are brain resident immune cells that maintain proteostasis and cellular homeostasis. Recent findings suggest that microglia dysfunction could contribute to the pathogenesis of Parkinson's disease (PD). One of the hallmarks of PD is the aggregation and accumulation of alpha‐synuclein (αSyn) into Lewy bodies inside nerve cells. Microglia may worsen the neuronal microenvironment by persistent inflammation, resulting in deficient clearing of aggregated αSyn. To model microglial behavior in PD, we utilized human induced pluripotent stem cells to generate functionally active microglia. We studied the microglial uptake of alpha‐synuclein preformed fibrils (PFFs) and the effect of pro‐inflammatory stimulation by interferon gamma. We demonstrate that combined exposure disrupts the phagosome maturation pathway while inflammatory stimuli suppress chaperone mediated autophagy and mitochondrial function. Furthermore, inflammatory stimulation impairs PFF uptake in microglia and increases cytokine production. Moreover, excessive PFF uptake by microglia results in induction of inducible nitric oxide synthase. Taken together, we demonstrate that this model is valuable for investigating the behavior of microglia in PD and provide new insights on how human microglia process aggregated αSyn.

## Main Points

- Inflammation exacerbates phagocytic clearance of αSyn PFFs in human microglia.
- Inflammatory environment impairs mitochondrial function while modulating protein degradation.

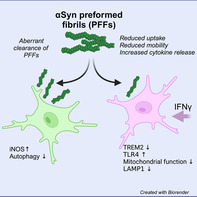

## 1 INTRODUCTION

Microglia are the resident innate immune cells of the brain which are involved in maintaining homeostasis and proteostasis in the central nervous system. They phagocytose pathogens, dying cells, cellular debris, and aggregated proteins. Microglia surveille the microenvironment and respond to both endogenous and exogenous signals with pro‐ or anti‐inflammatory functions. They can secrete soluble molecules, including cytokines and chemokines, supporting neuronal survival, neuro‐ and vasculogenesis, influencing permeability of the blood–brain barrier, or participating in inflammatory signaling (Bachiller et al., 2018; Li & Barres, 2018).

Current evidence suggests that microglia are implicated in the initiation or perpetuation of neuroinflammation, a phenomenon linked to many neurodegenerative diseases, including Parkinson's disease (PD) (Kwon & Koh, 2020; Muzio et al., 2021). Even though neurodegeneration involves the loss of neurons, the underlying pathogenic mechanisms may also be attributed to the non‐cell autonomous functions of glia rather than neurons themselves. Several inflammatory cytokines have been linked to PD (Reale et al., 2009; Tansey et al., 2022). One cytokine involved in inflammation is interferon gamma (IFNγ) which regulates immunological responses and strongly activates human microglia. It is secreted by T‐cells, NK cells, and in part by macrophages and monocytes (Darwich et al., 2009). Previous studies have indicated significantly increased levels of IFNγ in PD patient brains compared to control brains, particularly in the *substantia nigra* and *striatum* (Mogi et al., 2007). Moreover, in PD *substantia nigra* samples, a strong positive correlation was shown between IFNγ genes and *SNCA*, the gene encoding alpha‐synuclein (αSyn) (Liscovitch & French, 2014).

αSyn is a protein mainly localized in the presynaptic nerve terminals (Maroteaux et al., 1988; Stefanis, 2012) and its misfolding and aggregation into Lewy bodies is one of the hallmarks of PD (Spillantini et al., 1997). Microglia can uptake αSyn that has been released by neurons through several processes, such as phagocytosis (Zhang et al., 2005), selected autophagy or synucleinphagy (Choi et al., 2020), clathrin‐mediated endocytosis (Minami et al., 2012), and endocytosis facilitated by myosin‐7B and actin filaments (Zhang, Xu, et al., 2020). Selective synucleinphagy occurs when αSyn binds to TLR4, enters the cell, and activates the scavenger receptor p62/SQSTM1 via the NF‐κB mediated pathway (Choi et al., 2020). Notably, microglia can also share αSyn fibrillar load among themselves (Chakraborty et al., 2023; Scheiblich et al., 2021). Previous studies suggested that even though aggregated αSyn is rapidly taken up by the microglia, there is a delay in the degradation of the fibrils which resulted in accumulation of αSyn (Rostami et al., 2021; Scheiblich et al., 2021).

In terms of bioenergetics, microglia expend a lot of energy to perform key homeostatic functions. This energy is mostly provided by oxidative phosphorylation in the surveillance state; however, when inflammatory activation occurs, there is a metabolic switch toward aerobic glycolysis. Microglial functionality can be significantly impaired by mitochondrial dysfunction, as mitochondria play a crucial role in metabolic plasticity (Li et al., 2022). If microglia persistently engulf aggregated proteins and secrete proinflammatory factors, this excessive energy expenditure may disrupt key cellular processes.

Dysfunctional clearance of aggregated αSyn can potentially lead to spreading it to neighboring neurons. Additionally, microglia response to αSyn uptake can compromise their ability to process and degrade it, causing problematic accumulation (Grozdanov et al., 2019). A proposed contributor to PD pathogenesis is the ongoing release of proinflammatory factors by malfunctioning microglia. This may contribute to neuronal loss, and the reduced ability to clear away αSyn aggregates which results in a vicious cycle, further increasing neuroinflammation, protein deposition, and neurodegeneration. Currently, most studies on microglia function including exposure to aggregated forms of αSyn and induction of inflammation have been performed in murine cells (Bido et al., 2021). It is well‐established that there are species differences for example in the expression of PD‐related genes between mouse and human microglia (Gosselin et al., 2017). Therefore, to understand the human‐specific mechanism, we utilized an in vitro model of microglia derived from human induced pluripotent stem cells (hiPSCs). The cells were exposed to human αSyn preformed fibrils (PFFs) and IFNγ to elucidate how they process αSyn and whether this is affected in an inflammatory environment.

## 2 RESULTS

### 2.1 Differentiation of human iPSCs into maturated microglia and their characterization

**FIGURE 1:**
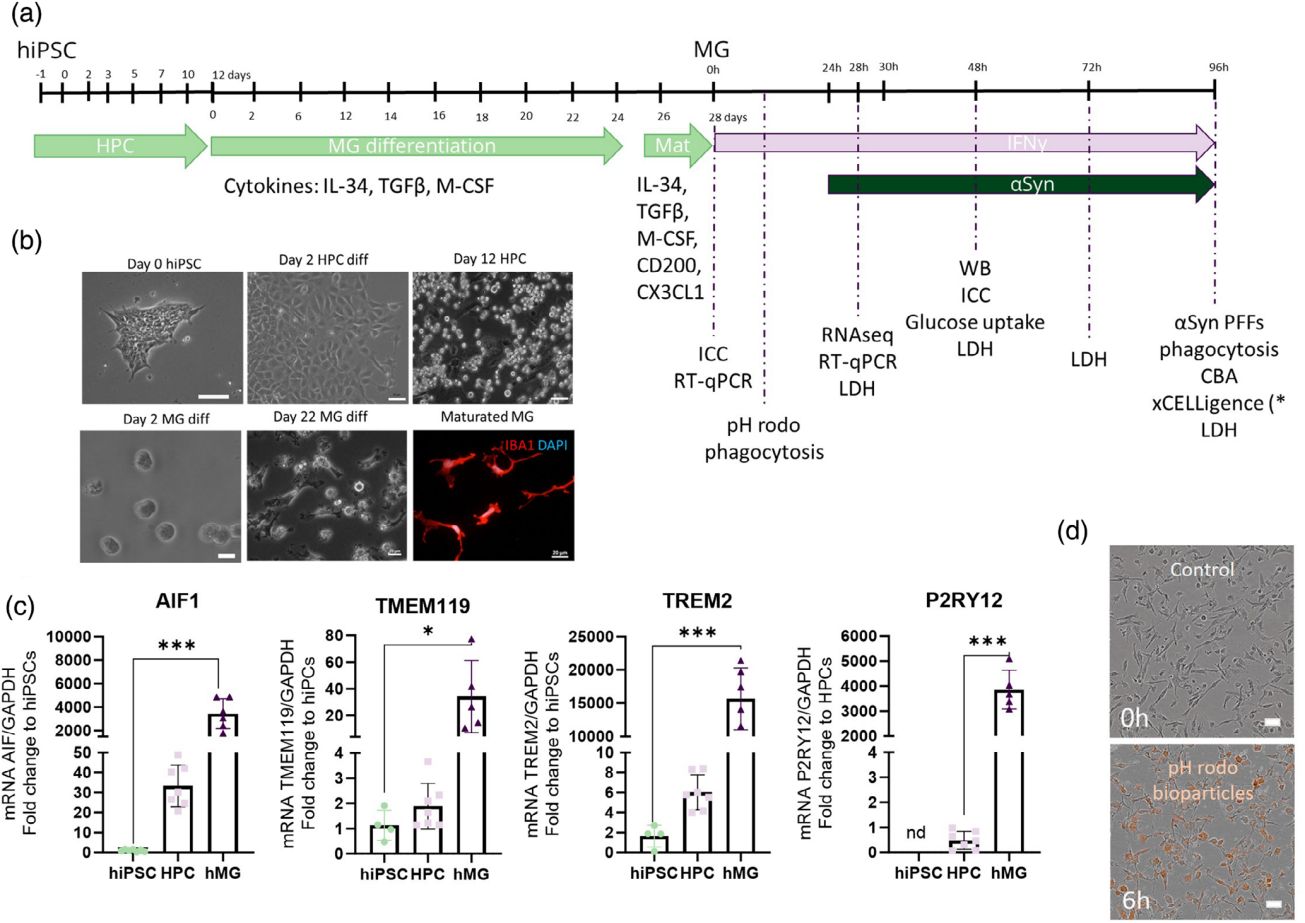
The hiPSCs differentiate into maturated human microglia cells with distinct microglial markers and functionality properties. (a) A timeline of microglia (hMG) differentiation and assay progression. The hMG are differentiated in a bi‐phasic protocol through hematopoiesis (HPC) and maturation before the assays were performed. Experimental setup, 24 h IFNγ 20 ng/mL pre‐treatment followed by 0.5 μM αSyn PFFs exposure. (b) Representative images along the differentiation process: Day 0 hiPSC, scalebar 100 μm. Day 2 HPC differentiation, scalebar 50 μm. Day 12 HPC formation, scalebar 50 μm. Second phase of hMG differentiation. Day 2, HPC plated for differentiation, scalebar 20 μm. Day 22 of hMG differentiation, scalebar 20 μm. Maturated hMG were stained with IBA1 and DAPI, scalebar 20 μm. (c) Gene expression of microglia‐specific markers AIF1, TMEM119, TREM2, and P2RY12 detected by RT‐qPCR from hiPSC, HPC, and hMG. Data normalized to GAPDH and presented as a fold change to hiPSC, except of P2RY12 to HPC (not detected [=nd]). (d) Representative images from microglia phagocytosing pHrodo bioconjugated particles (orange), from 0 h timepoint without beads to 6 h timepoint. Scalebar 50 μm. Data are presented as mean+/− SD. One‐way‐ANOVA was used to determine statistical differences. Tukey's multiple comparisons were used. HPC, hematopoietic progenitor cells; PFFs, alpha‐synuclein preformed fibrils. *p* < 0.05 = *, *p* < 0.01 = **, *p* < 0.001 = ***.

The microglia were generated from hiPSCs through hematopoietic progenitor cells (HPC) formation (Figure 1a) according to a modified protocol from Abud et al., 2017 and McQuade et al., 2018. In the experimental setup, we pre‐exposed half of the microglia to 24 h of IFNγ 20 ng/mL to mimic chronic inflammatory environment. To model PD, we then exposed another half of the cells to 0.5 μM αSyn PFFs. The dose was experimentally established (Appendix S1). The exposure groups were control, αSyn PFFs, IFNγ, and αSyn PFFs + IFNγ. The endpoint of the assay determined the fibril exposure time (mRNA collection 4 h PFFs exposure vs. 24 h PFFs exposure for protein collection) and can be seen in the timeline (Figure 1a). The cell lines used in this study are listed in a supplementary Table 1 (Appendix S1, Table 1) and specifications of which cell line in which assay Table 2 (Appendix S1, Table 2).

Maturated microglia exhibited a ramified structure and expressed microglia‐specific markers at both the transcriptional (RT‐qPCR) and translational levels (ICC). These markers included IBA1/AIF1 (Ionized calcium‐binding adaptor molecule 1/Allograft inflammatory factor 1; microglia/macrophage marker), TMEM119 (Transmembrane Protein 119; mature microglia‐specific marker, not expressed by macrophages), TREM2 (Triggering receptor expressed on myeloid cells 2; microglia surface receptor), and P2RY12 (Purinergic Receptor P2Y12; homeostatic microglia marker) (Figure 1b,c, Appendix S1, Figure 1a). When compared to hiPSCs, the gene expression of these markers (*AIF1*, *TREM2*, *TMEM119*) was significantly increased, indicating effective differentiation into microglia. To assess the microglial capacity of phagocytosis, bioparticles containing pH‐dependent dye were used. pHrodo beads become fluorescent once a phagosome merges with a lysosome, the pH drops below 5 and activates the fluorescence within the beads. The observed phagocytic process by human microglia is fast, and within minutes the fluorescence is detected. Representative images shown (Figure 1d). These assays indicate that the human microglia generated are maturated and functioning as expected.

### 2.2 Transcriptomic analysis of human microglia exposed to alpha‐synuclein preformed fibrils and interferon‐gamma shows changes in several key pathways related to microglial function

**FIGURE 2:**
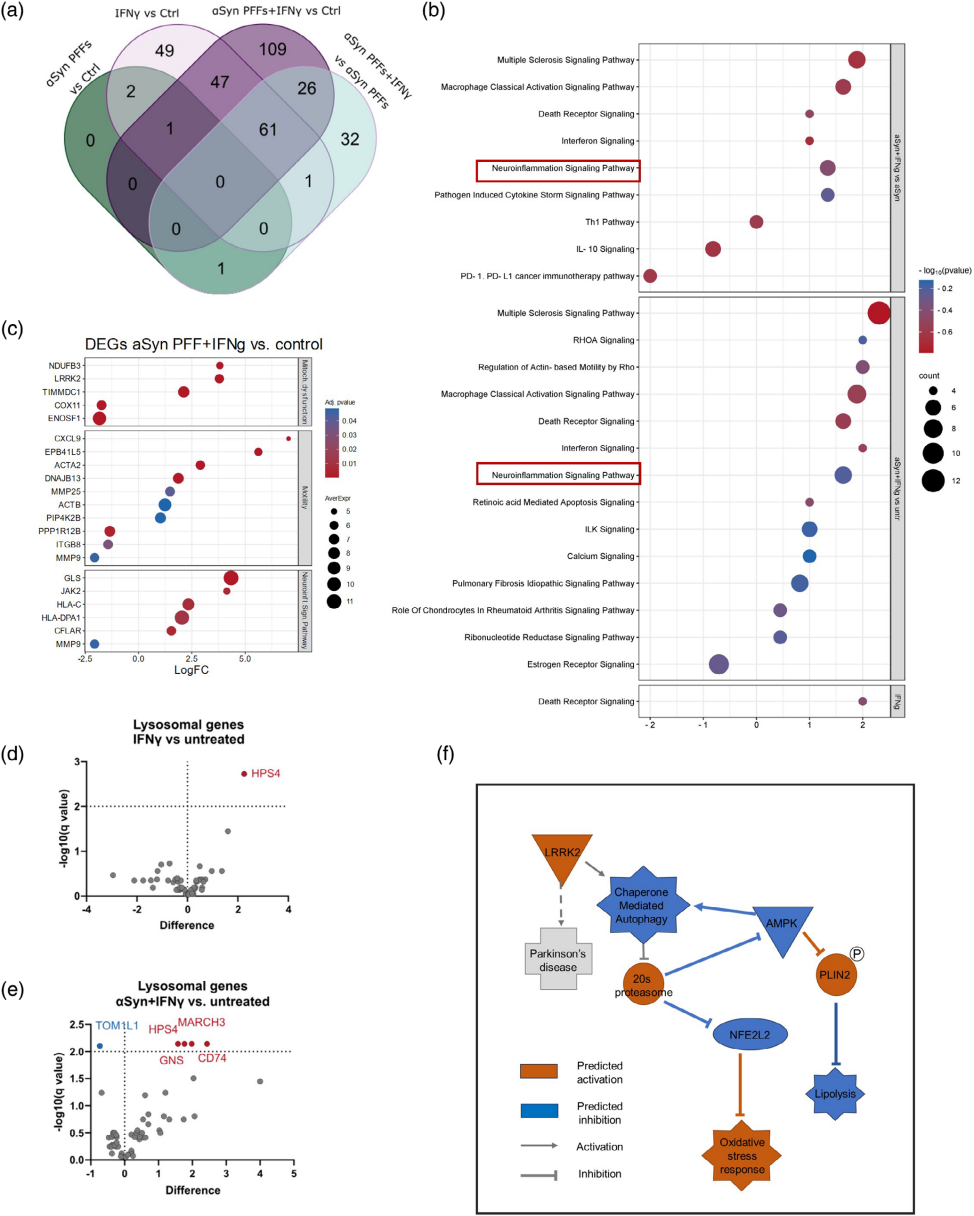
Transcriptomics reveal microglial inflammatory activation. (a) Venn diagram of differentially expressed genes (DEGs) from different comparison groups. (b) A bubble plot from the statistically significant, activated, or inhibited pathways discovered from the IPA, the z‐score indicating pathway activation (positive value) or inhibition (negative value). Comparison groups: alpha‐synuclein (αSyn) PFFs + IFNγ vs. αSyn PFFs, αSyn PFFs + IFNγ vs. Control, IFNγ vs. Control. (c) DEGs associated with activation of neuroinflammation signaling pathway, mitochondrial dysfunction, and motility from the comparison group αSyn PFFs + IFNγ vs. Control. Volcano plots from Lysosomal genes in the comparison groups; (d) IFNγ vs. control (untreated) and (e) αSyn PFFs + IFNγ vs. control (untreated). Multiple unpaired t‐tests utilized. Genes upregulated (red) and downregulated (blue). (f) αSyn degradation pathway alterations comparing the αSyn PFFs + IFNγ to αSyn PFFs reveal the effect of inflammation on gene expression on this pathway. IPA, ingenuity pathway analysis; PFFs, alpha‐synuclein preformed fibrils.

RNA sequencing (RNAseq) was used to investigate the impact of exposure to αSyn PFFs and IFNγ on the transcriptome of human microglia. Cells were pre‐exposed to IFNγ for 24 h and then cultured for an additional 4 h with or without αSyn PFFs. The exposure time was experimentally established (see section 4, *Bulk RNAseq*). A pairwise analysis identified four differentially expressed genes (DEGs) between αSyn PFF exposed microglia and control, while IFNγ exposure altered 161 DEGs (Figure 2a). Comparing αSyn PFF + IFNγ to the control cells revealed 244 DEGs. Overall, the gene expression signatures of the IFNγ and αSyn PFF + IFNγ groups were closely clustered together, whereas the control and αSyn PFF groups showed similarities (Appendix S1, Figure 2).

**FIGURE 3:**
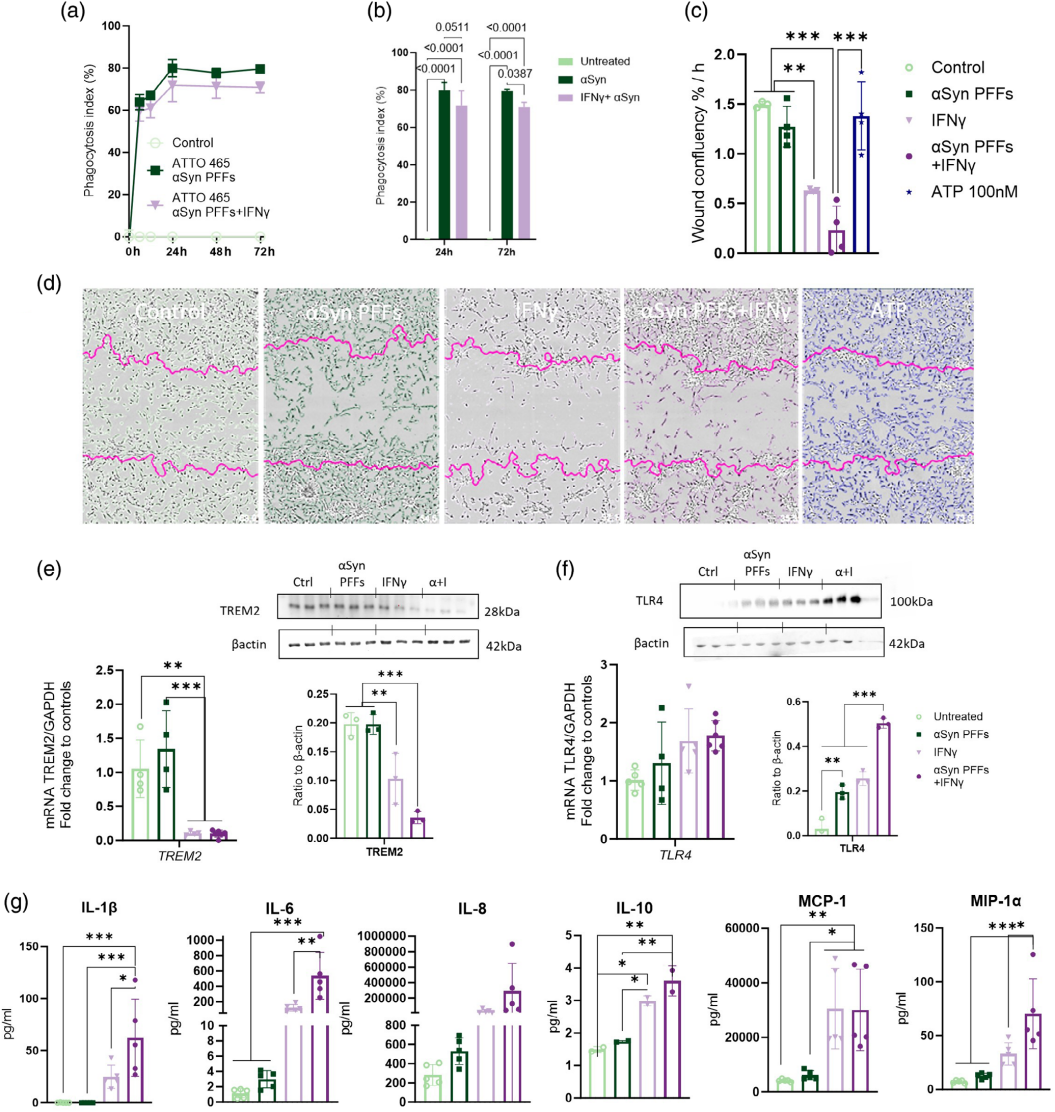
Microglial phagocytosis of alpha‐synuclein (αSyn) PFFs and cytokine release is affected by the inflammatory environment (a) Live phagocytosis imaging of 0.5 μM ATTO465‐labeled αSyn PFFs –monitored for 72 h. Microglia were exposed to IFNγ 20 ng/mL for 24 h prior to imaging. (*n* = 3 wells/treatment, 2 images/well) (b) The statistics for ATTO465‐labeled αSyn PFFs phagocytosis at timepoint 24 and 72 h. (c) Scratch wound assay was utilized to assess the microglial mobility, movement speed upon wound closure analyzed. Cells were exposed to IFNγ 20 ng/mL (where applicable) for 24 h, then the αSyn PFFs 0.5 μM and ATP 100 nM were added at the start of the assay. (d) Representative images from scratch wound assay at 24 h timepoint. Images color coded to match the graph. Gene expression and protein quantification of (e) TREM2 and (f) TLR4. RT‐qPCR after 4 h 0.5 μM αSyn PFFs exposure, western blot 24 h exposure to 0.5 μM αSyn PFFs with 28/48 h exposure to IFNγ 20 ng/mL. (g) Cytokine bead array (CBA) was used to determine the microglial cytokine secretion into the media. Microglia were pre‐stimulated with IFNγ 20 ng/mL for 24 h before adding 0.5 μM αSyn PFFs for 72 h. Statistics on all graphs one‐way ANOVA with Tukey's multiple comparisons test. PFFs, alpha‐synuclein preformed fibrils. *p* < 0.05 = *, *p* < 0.01 = **, *p* < 0.001 = ***. Data are presented as mean +/− SD.

**FIGURE 4:**
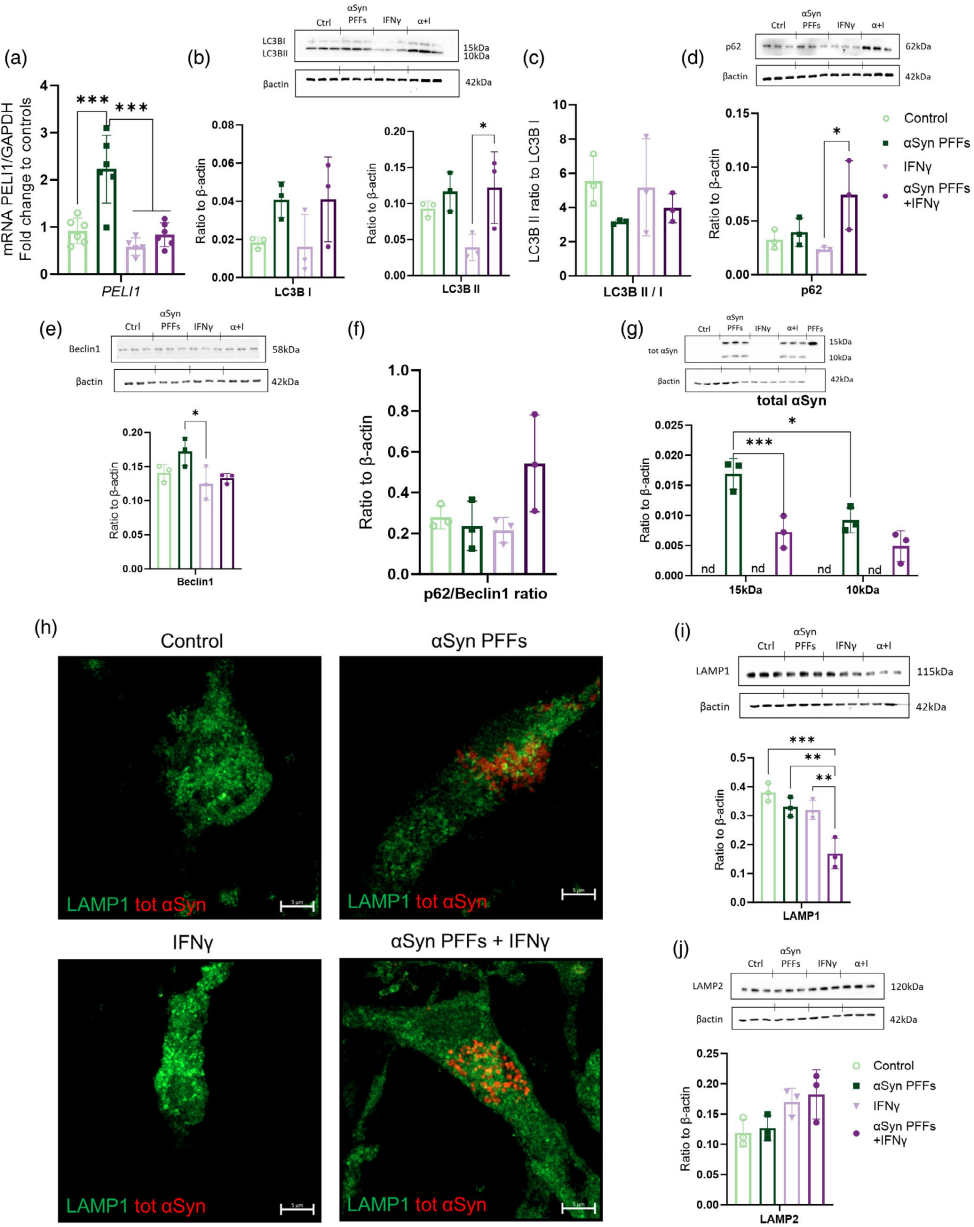
Microglial processing of alpha‐synuclein (αSyn) PFFs is affected by the inflammatory environment. (a) Gene expression of PELI1 (αSyn PFFs 4 h + IFNγ 28 h). (b) Protein expression of autophagosomal protein LC3B, with quantifications. (All protein analyses depicted had exposure of αSyn PFFs 24 h + 48 h IFNγ) (c) The ratio between LC3BII to LC3BI expressed proteins. Protein analysis of autophagosome formation markers (d) p62 and (e) Beclin1 with their ratio (f). (g) Protein expression of total αSyn within the cells. Control PFFs—not in contact with the cells—were used to normalize the data. (nd = not detected) (g) LAMP1 (green) and tot αSyn (red) staining on exposed microglia (αSyn PFFs 24 h and IFNγ 48 h). Scale bar 5 μm. Western blot from lysosomal associated membrane proteins (j) LAMP1 and (k) LAMP2. Data are presented as mean +/− SD. Statistics one‐way‐ANOVA (F = two‐way‐ANOVA) with Tukey's multiple comparisons test. PFFs, alpha‐synuclein preformed fibrils. *p* < 0.05 = *, *p* < 0.01 = **, *p* < 0.001 = ***.

**FIGURE 5:**
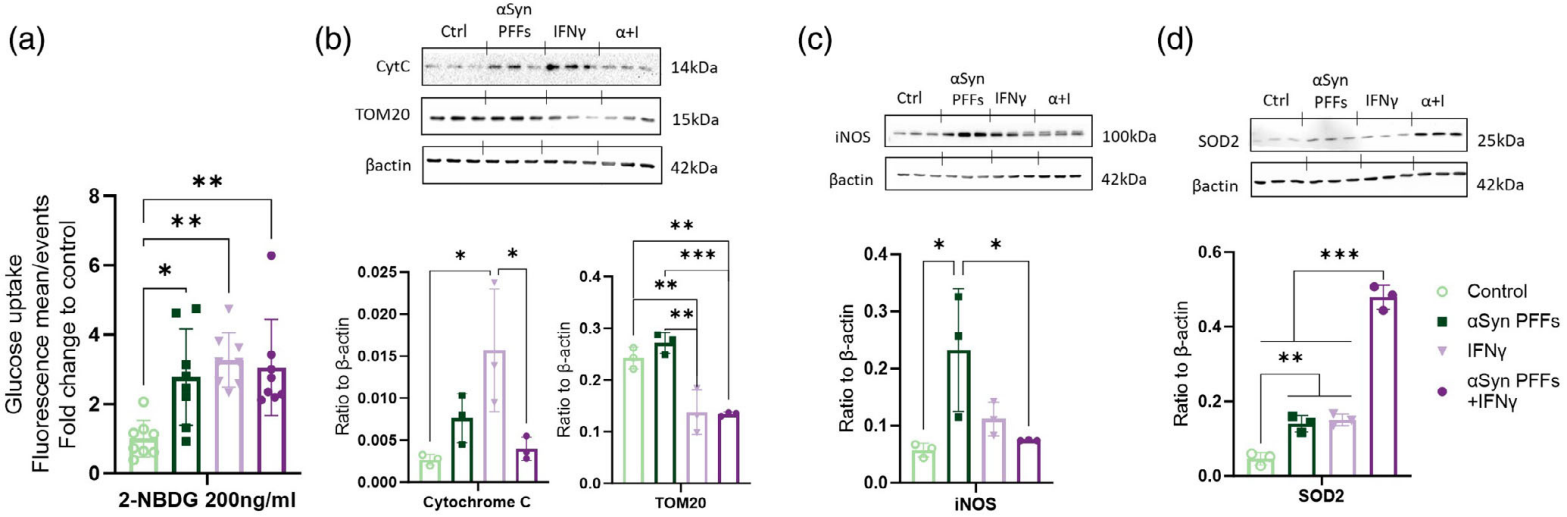
Microglia exposed to alpha‐synuclein (αSyn) PFFs show signs of metabolic changes and mitochondrial stress when combined with inflammatory environment. (a) Glucose uptake analyzed with 200 ng/mL 2‐NBDG, a labeled glucose, detected on the FITC‐A channel with flow cytometer. WB from (b) mitochondrial markers Cytochrome C and TOM20, (c) inducible nitric oxide synthase (iNOS), (d) and mitochondrial superoxide dismutase (SOD2), with quantifications. Data are presented as mean +/− SD. Statistics one‐way ANOVA with Tukey's multiple comparisons test. iNOS, inducible nitric oxide synthase; PFFs, alpha‐synuclein preformed fibrils. *p* < 0.05 = *, *p* < 0.01 = **, *p* < 0.001 = ***.

An analysis of the ingenuity pathway analysis (IPA) revealed activation of neuroinflammation signaling pathway in the comparison groups of αSyn PFFs + IFNγ versus control and αSyn PFFs + IFNγ to αSyn PFFs (Figure 2b, Appendix S1, Figures 3 and 4). This finding provides evidence that IFNγ exposure alone is insufficient to simulate the neuroinflammatory phenotype (Appendix S1, Figure 5). Specifically, *GLS*, *JAK2*, and human leukocyte antigen (HLA) genes *HLA‐C*, *‐DPA1*, and *‐DRA were* significantly upregulated in the neuroinflammation signaling pathway compared to control cells exposed to αSyn PFFs and IFNγ alone. *MMP9* was significantly downregulated (Figure 2c). Further, IPA detected dysregulation in the pathway associated with mitochondrial dysfunction. In the comparison αSyn PFFs + IFNγ to control the genes *LRRK2*, *NDUFB3*, and *TIMMDC1* were upregulated, while *COX11* and *ENOSF1* were downregulated. Several genes related to microglia motility were also upregulated in the αSyn PFFs + IFNγ group compared to the control. These genes include *ACTA2*, *ACTB*, *CXCL9*, *DNAJB13*, *EPB41L5*, *ITGB8*, *MMP25*, *MMP9*, *PIP4K2B*, and *PPP1R12B*.

Regarding the processing of the αSyn PFFs, both the IPA pathways and lysosomal DEGs were inspected. The combined exposure to αSyn PFFs and IFNγ was disrupting the phagosome maturation pathway, particularly by upregulating lysosomal genes *HPS4*, *MARCH3*, *GNS*, *CD74* and downregulating *TOM1L* (Figure 2e). On the other hand, IFNγ exposure alone only increased the expression of *HPS4* (Figure 2d). The effect of IFNγ to the αSyn degradation was demonstrated by comparing αSyn PFFs + IFNγ to αSyn PFFs (Figure 2f). The inflammatory stimuli suppressed chaperone mediated autophagy, AMPK, and NFE2L2 while activating LRKK2, 20s proteasome, and PLIN2. Based on the RNAseq findings, we investigated the impact of the exposures on the human microglia further at the functional and protein levels.

### 2.3 A proinflammatory environment impairs phagocytosis of alpha‐synuclein preformed fibrils and increases cytokine release in human microglia

To elucidate how the inflammatory environment affects the phagocytosis of αSyn PFFs, the cells were again pre‐exposed with IFNγ for 24 h. Following this exposure, the phagocytosis assay was initiated with ATTO 465‐labeled αSyn PFFs. The labeled PFFs were rapidly engulfed in the first few hours. At the 6 h timepoint, approximately 64% of cells showed significant fluorescence from the αSyn PFFs, in contrast to only 60% of the αSyn PFFs + IFNγ exposed group (Figure 3a, Appendix S1, Figure 1f). At 24 h the phagocytosis reached a plateau and changes in the cells exposed to IFNγ started to emerge (Figure 3b). 80% of the αSyn PFF‐exposed compared to 72% of the αSyn PFFs + IFNγ exposed, had remarkably ingested ATTO‐labeled αSyn PFFs and were found fluorescent. The fluorescence emitted by the labeled PFFs remains detectable even after 72 h and the number of fluorescent cells decreased only by 0.5% (αSyn PFFs) or 1% (αSyn PFFs + IFNγ) from the 24 h timepoint. A statistically significant reduction of αSyn PFF phagocytosis was observed after IFNγ (−9%) at timepoint 72 h (Figure 3b).

A scratch wound assay was performed to assess microglial migration and its potential impact on phagocytosis (Figure 3c,d). ATP 100 nM was used as a positive control, and cells were exposed to IFNγ 24 h prior. The administration of αSyn PFFs alone resulted in a modest decrease in mobility speed compared to the control group at the 24 h timepoint (Figure 3c). IFNγ reduced microglial movement, resulting to inefficient wound base refilling by microglia. Exposure to αSyn PFFs + IFNγ resulted in decreased movement speed. Both immunostimulated groups covered less than half of the wound base compared to ATP‐stimulated microglia (Figure 3d). To look at the kinetics of the internal response, or motility, of microglia, bioimpedance measurement was conducted (Appendix S1, Figure 7b and c). αSyn PFFs alone had no impact on cellular impedance, resulting in a comparable curve to the control. IFNγ increased the cellular impedance.

Next, we wanted to study how the cell surface receptors that initiate phagocytosis were changed in response to the treatments. Gene expression of triggering receptor expressed on myeloid cells 2 (*TREM2*) (Figure 3e) and toll‐like receptor 4 (*TLR4*) (Figure 3f) were analyzed 4 h post‐αSyn PFFs exposure (with or without 24 h pre‐exposure to IFNγ) with RT‐qPCR. The mRNA expression of *TREM2*, a known enhancer of phagocytosis, was decreased significantly after IFNγ stimulus. mRNA of *TLR4* was not significantly changed with any of the conditions, although some groups of cells showed a clear upregulation. To validate these results at the protein level, we used western blot at 24 h post‐αSyn PFF exposure when the phagocytosis plateaued. Similarly to the gene expression results, TREM2 levels decreased significantly upon IFNγ exposure. In contrast, TLR4 levels increased significantly. The αSyn PFFs + IFNγ caused an almost 5‐fold increase of TLR4 from the control and 4‐fold decrease of TREM2.

To study the neuroinflammatory signaling, the microglial cytokine release was analyzed with CBA assay using medium collected 72 h post‐αSyn PFFs exposure (Figure 3g). The αSyn PFFs alone did not induce statistically significant alterations. The microglia exposed to inflammatory stimulus with or without αSyn PFFs increased greatly the secretion of cytokines IL‐1β, IL‐6, IL‐8, IL‐10, MCP‐1, and MIP‐1α. However, a synergistic effect of αSyn PFFs exposure with IFNγ pre‐stimulation resulted in increased release of IL‐1β (2.5‐fold), IL‐6 (4.4‐fold), IL‐8 (9.3‐fold), and MIP‐1α (1.8‐fold increase) compared to the cells stimulated solely by IFNγ.

### 2.4 Alpha‐synuclein preformed fibril clearance is inhibited in pro‐inflammatory microglia

In order to investigate the clearance of the internalized αSyn PFFs, we studied the proteins related to autophagy. Based on previous literature, we selected a few markers relevant for autophagy. We assessed the expression level of *Pellino E3 Ubiquitin Protein Ligase1* (*PELI1*), a gene known to participate in the ubiquitin‐proteasome system. We could see a trend in the RNAseq results and wanted to confirm this with repeat RT‐qPCR measure. We observed a significant upregulation of PELI1 upon αSyn PFFs exposure (Figure 4a). Next, as an autophagy marker we examined the LC3B subtypes I and II, and their ratio (Figure 4b,c). Microtubule‐associated protein LC3 has different isoforms, LC3BI is the cytosolic form, whereas the lipidated and conjugated form LC3BII marks the formation of complete autophagosome. The αSyn PFFs induced a drop in LC3B subtype II to subtype I ratio, whereas IFNγ was reducing the LC3B protein. We also examined the protein levels of selective autophagosome formation marker p62 (Figure 4d) and autophagy regulator Beclin1 (Figure 4f). There was a sharp rise in p62 in IFNγ stimulated and αSyn PFFs exposed microglia.

The p62/Beclin1 ratio also was increased in these cells (Figure 4h), indicating autophagy impairment upon inflammatory environment.

Control microglia do not have detectable levels of endogenously expressed αSyn (Figure 4g, control, Figure 4h, control). After 24 h, αSyn PFFs exposed microglia, when assessed with western blot, showed prominent intracellular levels of αSyn at size 15 kDa. A significantly lower amount of αSyn protein was detected in the cells exposed to both αSyn PFFs and IFNγ, confirming that the microglia phagocytose fewer αSyn PFFs under inflammatory conditions. In addition to the expected 15 kDa fragment, microglia processed the αSyn PFFs into a 10 kDa fragment.

To investigate effects on the lysosomal system, microglia were stained for both total αSyn and lysosomal associated membrane protein 1 (LAMP1) (Figure 4h). αSyn puncta were exclusively observed in the microglia treated with PFFs. Additionally, IFNγ induced a morphological change in the outer membrane of microglia, resulting in spindle‐like morphology (Figure 4h, Appendix S1, Figure 1f). The level of LAMP1 was significantly decreased when microglia were simultaneously exposed to the αSyn PFFs and IFNγ (Figure 4i). The LAMP2 levels, however, had a slight tendency to increase with IFNγ exposure, but there was no significant change seen (Figure 4k).

### 2.5 Uptake of alpha‐synuclein preformed fibrils by human microglia induces an aberrant phenotype associated with reduced mitochondrial function

In addition to analysis of uptake functions of the human microglia after exposure to αSyn PFFs and inflammation induction, we explored impact on energy metabolism to assess the mitochondrial dysfunction. We found that glucose uptake was increased, indicating metabolic switch to glycolytic phenotype which is also related to inflammatory response (Lauro & Limatola, 2020). There was an approximately 3‐fold increase when cells were exposed to αSyn PFFs and/or IFNγ (Figure 5a). IFNγ exposure significantly reduced mitochondrial content in human microglia assessed by TOM20 (translocase of outer mitochondrial membrane 20) (Figure 5b). In contrast, the Cytochrome C content significantly increased (Figure 5b). Cytochrome C is the electron shuttling protein involved in synthesis of ATP, located in the inner mitochondrial membrane. αSyn PFFs alone strongly increased inducible nitric oxide synthase (iNOS) expression, not evident upon IFNγ stimulation (Figure 5c). Additionally, superoxide dismutase 2 (SOD2), that processes reactive oxygen species in the mitochondrial matrix, was significantly increased in all exposures, where addition of the αSyn PFFs to IFNγ exposed microglia potentiated this increase 3.1‐fold (Figure 5d).

## 3 DISCUSSION

Disturbed CNS proteostasis is considered a key feature in the pathophysiology of PD (Lehtonen et al., 2019), and one of the main functions of microglia is to clear away debris, including protein aggregates such as αSyn. To untangle the effects of how microglia respond to increased levels of αSyn, we set up an in vitro model consisting of hiPSC‐derived microglia and exposed them to human αSyn PFFs. Since neuroinflammation is also thought to contribute to PD pathogenesis, we further exposed the hiPSC‐derived microglia to the physiologically relevant cytokine IFNγ, shown to be elevated in PD brains (Mogi et al., 2007) and primarily secreted by non‐microglial cells (Billiau & Matthys, 2009), and investigated how this affected αSyn processing and clearance.

hiPSC‐derived microglia expressed markers such as TREM2, TMEM119, and P2RY12, matching to the microglia analyzed from human post mortem samples in a single‐cell mass cytometry study (Böttcher et al., 2019). The maturated human microglia phagocytosed particles on their own without any exposure, indicating them to be functionally active.

In this study, we wanted to explore the effects of fibrillar αSyn on human microglia. Exposing neurons to αSyn PFFs results in Lewy body formation that resembles the ones observed in patients (Mahul‐Mellier et al., 2020). In contrast, monomeric αSyn does not seed endogenous αSyn into aggregates (Volpicelli‐Daley et al., 2011) and is not taken up by primary mouse microglia (Hoffmann et al., 2016). For these reasons, we utilized the αSyn PFFs as part of our model.

RNAseq of the transcriptome of the human microglia exposed to αSyn PFFs in the inflammatory environment induced activation of the neuroinflammation signaling pathway; interestingly, this did not occur with the IFNγ exposure by itself. This provides further evidence to the concept that neuroinflammation is caused by both an inflammatory environment and the presence of fibrillar aggregates. Several key microglial signaling molecules were synergistically elevated in the functional assays. Another important finding from this data is the upregulation of HLA genes when microglia were exposed to both fibrillar αSyn and IFNγ. HLA‐DR microglia have been found in PD patient *substantia nigra* (McGeer et al., 1988) and this may indicate antigen presentation initialized by αSyn. Other changed genes and pathways generated from this data led us to study further the effects in the human microglia upon these exposures.

We observed inhibition of phagocytosis of αSyn PFFs by human microglia in the inflammatory environment, as evidenced by fewer cells taking up the labeled fibrils. This reduced uptake was most likely due to the decreased mobility of the microglia exposed to IFNγ, as they were clearly less mobile in the scratch wound assay. A previous study indicated that fibrillar αSyn gets taken into hiPSC‐derived macrophages through actin‐dependent phagocytosis pathways (Haenseler et al., 2017), and perhaps this is inhibited due to inflammation. Our results are in concert with the phagocytosis results of Ohtonen et al., 2023 where IFNγ was also seen to decrease microglial phagocytosis in hiPSC‐derived PD and control microglia (Ohtonen et al., 2023).

In connection with the observed suppression of phagocytosis of αSyn PFFs after induction of inflammation, we show a decrease in TREM2 and an increase in TLR4. This is in line with previous research since in primary mouse microglia, TREM2 has been shown to promote phagocytosis in microglia (Hsieh et al., 2009) and TLR4 was necessary for the phagocytosis of αSyn species (Fellner et al., 2013; Venezia et al., 2021). The role of TLR4 is still somewhat controversial as some studies indicate that TLR4 activation through αSyn inhibits microglial autophagy (Brockmann et al., 2016). Interestingly, with the addition of the αSyn PFFs after the inflammatory stimulation, we observed synergetic effects in the potentiation of the microglial inflammatory phenotype that was not as prominent with either of the exposures alone. The primed microglia responded to the fibrils with enhanced excretion of cytokines and chemokines. The chemokines that were elevated by the combination of αSyn PFFs together with IFNγ—IL‐1β, IL‐6, and IL‐10—were also seen elevated in the serum samples from patients diagnosed with idiopathic PD (Brockmann et al., 2016). IL‐1β and IL‐6 may recruit even more IFNγ‐producing cells (T‐cells, NK cells, and macrophages) into the vicinity and reinforce the proinflammatory microenvironment (Bido et al., 2021), whereas IL‐10 has a protective, anti‐inflammatory effect. We saw an increase in the secretion of MIP1α, a strong chemotactic molecule attracting, for example, T‐cells, upon IFNγ, that was potentiated with the addition of αSyn PFFs as well. CD4^+^ and CD8^+^ T cells have been observed in PD post mortem brains and further, an important study by Sulzer et al., 2017 indicated that CD4^+^ T cells from PD patients recognize certain αSyn‐derived peptides (Sulzer et al., 2017). This is one way in which the peripheral immune system may initiate or exacerbate neuroinflammation in the brains of PD patients. Our data strengthen the concept that release of cytokines by reactive microglia is further enhanced in the presence of excessive amounts of αSyn, which may lead to infiltration of peripheral immune cells. However, it is still not clear which event comes first to trigger a disease state; although evidence suggests that non‐motor symptoms such as gastrointestinal dysfunction related to inflammation precede the diagnosis of PD (Weller et al., 2005). Here, when human microglia were primed with an inflammatory stimulus and exposed to αSyn PFFs, even though they phagocytosed fewer labeled particles, the added presence of αSyn still resulted in increased cytokine release. This suggests that both need to be present to create a detrimental inflammatory environment.

Interestingly, we show that the human microglia are breaking down the uptaken αSyn into a smaller 10 kDa fragment. Additionally, the microglia retain the labeled αSyn inside after 3 days as demonstrated in the phagocytosis assay. It has also been shown that microglia from hiPSCs continue to have labeled αSyn after 7 days post‐exposure to αSyn PFFs (Rostami et al., 2021), indicating a delay or prolonged processing taking place. To elucidate more how the human microglia process αSyn, we analyzed autophagy and lysosomal protein levels. We observe a clear increase in *PELI1* expression when the microglia are exposed to the αSyn PFFs alone. The observed αSyn PFFs induced a drop in LC3B subtype II to subtype I ratio indicating decreased autophagosome formation may be caused by rise in PELI1 expression, known to impair autophagy (Guo et al., 2020). The *PELI1* has also a suggested role as the mediator of neuroinflammation and microglia activator through regulation TLR pathway (Xiao et al., 2013).

Upon IFNγ exposure, when PFF phagocytosis is reduced, intracellular amount of full length and fragmented αSyn decreases, coinciding with significant elevation of p62 and reduction in LAMP1 levels which may indicate lysosomal deficiency leading to inhibited PFF clearance. and no change from control with IFNγ. PELI1 is involved in substrate ubiquitination and most interestingly, has been found to be highly expressed in microglia and was required for their activation (Xiao et al., 2013). Given that selective synucleinphagy is driven by TLR4 and downstream activation of p62, we could hypothesize that this is the pathway activated here, and further that it requires a large amount of substrate to become noticeably upregulated. A decrease in LAMP1 protein level was shown in primary mouse cells overexpressing αSyn (Rojanathammanee et al., 2011). We did not detect such a change in our human microglia with αSyn PFFs alone, but only when the cells were exposed to IFNγ together with the αSyn PFFs. Its decrease in an inflammatory environment may suggest that the lysosomal degradation pathway is not fully functioning in such a case. This further contributes to the existing knowledge of dysfunctional protein clearance via microglia in a proinflammatory environment.

The outcomes of this on microglia energy dynamics resulted in increased glucose uptake in cells exposed to IFNγ accompanied by decreased mitochondria content, indicating an elevation in glycolysis. There is evidence of inflammation causing dysfunction in mitochondria (López‐Armada et al., 2013). However, the inflammatory environment significantly increases cytochrome C content, likely to support respiration required for proper IFNγ signaling. Consistent with previous research, it has been observed that in antigen presenting cells IFNγ response requires mitochondrial respiration. Furthermore, these cells are unable to activate T cells when complex I is genetically or chemically inhibited (Kiritsy et al., 2021). Transcriptomic pathway analysis hints toward a dampened oxidative stress response in the immunostimulated phagocytosing group. In contrast, αSyn PFFs alone strongly induce iNOS expression, which is not happening in IFNγ‐exposed microglia. Additionally, they suppress the activation of cytochrome C and lead to the mitochondrial potential collapse upon induction of an inflammatory environment. Both exposure to αSyn PFFs and IFNγ alone resulted in a clear increase in SOD2 protein expression. This rise was further enhanced upon addition of αSyn PFFs to the inflammatory environment. SOD2 is responsible for scavenging superoxide radicals produced during respiration. It was shown that SOD2‐deficient mice had worsened αSyn pathology compared to wild‐type littermates (Scudamore & Ciossek, 2018). This implicates SOD2 is directly responsible for managing αSyn‐related mitochondria damage and goes along with our results from other mitochondrial proteins. Altogether the results suggest that αSyn PFF uptake compromises the ability of human microglia to properly respond to activation and indicates phagocytic exhaustion, potentially causing mitochondrial dysfunction.

In this study, we have utilized hiPSC‐derived microglia to examine effects of fibrillar αSyn on these cells, particularly in an inflammatory environment. We demonstrate complementary results in how human microglia respond to and manage αSyn load, as well as downstream effects on key cellular processes such as autophagy and energy metabolism. Further, we show how this is affected when inflammation is present in order to mimic a proposed pathophysiologic event in PD. We suggest this is a useful model to dissect microglial response to αSyn in various conditions.

## 4 MATERIALS AND METHODS

### 4.1 Culturing human iPSCs

The human iPSCs from healthy donors were previously generated and characterized lines (Fagerlund et al., 2021; Oksanen et al., 2017), together with commercial lines (Takara Bio Y00275 and Y00325) (Appendix S1 Table S1). The patient‐derived material has the approval of the Hospital District of Northern Savo Research Ethics Committee (#42/2010 and #123/2016). Visual monitoring of the cells and changing of Essential 8 hES cell culture media (Life Technologies, A1517001) with Penicillin/Streptomycin (Invitrogen, 5140–122) was performed daily to ensure quality of the cells. The cells were split using 0.5 mM EDTA (Life Technologies, 15,575), and grown on Matrigel (Corning, 356,231)‐coated dishes.

### 4.2 Differentiating microglia from human iPSCs

The protocol has been optimized from previously published protocols (Abud et al., 2017; McQuade et al., 2018). In short, the hiPSCs were cultured for approximately 10 days and split every few days. At confluency 60%–75% the colonies were detached using ReLeSR (StemCell Technologies, 05872). Then approximately 14–30 colonies were plated on Matrigel‐coated 6‐well plates (Greiner bio‐one, 6571690). The next day, the wells with approximately 16 to 20 colonies were chosen for culturing the HPCs. Hematopoietic Kit (StemCell Technologies, 05310) was used according to the manufacturer's protocol. The floating cells were harvested between days 11 and 16 of the differentiation and plated on Matrigel‐coated dishes for microglia differentiation.

Microglia differentiation lasted 25 days (±3 days). The cells were fed by adding 1:3 of fresh microglia culturing media (MG‐media) every other day. MG‐media consists of DMEM/F12 (Gibco, 11320033), Insulin transferrin (Gibco, 41400045), B27 (Gibco, 17504001), MEM Non‐Essential Amino Acids Solution (100×, Gibco, 11140050), GlutaMax (Gibco, 35050061), Penicillin/Streptomycin 10,000 U/mL (Gibco, 15140122), N2 (Gibco, 17502001), Human Insulin (Sigma‐Aldrich, I9278), and 1 M 1‐Thioglyserol (Sigma‐Aldrich, M1753). The differentiating cytokines used were IL34 (100 ng/mL, SinoBiological, 158‐10,948‐H08S‐100), TGFβ (50 ng/mL, Peprotech, 100–21), and M‐CSF (25 ng/mL, Peprotech, 300–25). On day 12 of the differentiation, the medium was reduced (leaving old media and adding fresh media on top, 1:3), or the cells were divided into two dishes/wells with the addition of fresh media (1:3). For the maturation, the cells were detached with Accutase (StemCell Technologies, 07920) and plated on the assay plates in the desired density. The 3‐day maturation was done by initially adding 50% differentiation media (collected at time of harvest) and 50% maturation media with IL34 (100 ng/mL), TGFβ (50 ng/mL), M‐CSF (25 ng/mL), CD200 (100 ng/mL, Biolegend, 770,004), and CX3CL1 (100 ng/mL, Peprotech, 300–31). For the assays, MG‐media with only IL34 (100 ng/mL), and M‐CSF (25 ng/mL), was used.

### 4.3 Immunocytochemistry

Cells were plated to Matrigel‐coated 24‐well plates with glass using 60,000 cells/well. Cells were either non exposed (characterization) or exposed 24 h to αSyn PFFs and or 48 h IFNγ. The cells were fixed with 4% PFA for 20 min at room temperature (RT). Cells were washed 5× with PBS, permeabilized with 0.2% Triton‐X at RT for 30 min, and blocked with 5% normal goat serum or 5% horse serum in PBS (blocking buffer) for 1 h at RT. Cells were incubated with the primary antibodies Iba1 (1:400, Wako, 019–1974), P2RY12 (1:125, Sigma, HPA14518), TMEM119 (1:100, Abcam, ab18533), CX3CR1 (1:200, Abcam, ab8021), LAMP1 (1:500, Novus Biologicals, NBP2‐25154SS), total αSyn (1:500, Abcam, ab138501) in blocking buffer incubated overnight at 4°C. The next day, cells were washed 5× with PBS and incubated with secondary antibodies 1:300 in PBS goat anti‐rabbit Alexa fluor 488 (Invitrogen antibodies, A11008), goat anti‐rabbit Alexa fluor 568 (Invitrogen Antibodies, A11011) for 1 h at RT (dark conditions), then washed again 5× with PBS. Nuclei were stained with DAPI in PBS (1:2000, Sigma, D9542) for 5 min at RT. Cells were then washed 4× PBS and 1x MilliQ. Coverslips were mounted on glass slides (Thermo Scientific, J1800AMNZ) with Fluoromount G (Southern Biotech, 0100–01). The images were taken with Zeiss Imager AX10 (characterization) and Zeiss LSM 800 with Airyscan (αSyn PFFs exposures).

### 4.4 LDH cytotoxicity assay

To assess the toxicity of the concentration of αSyn PFFs (0–1 μM), and combination of αSyn PFFs 0.5 μM + IFNγ 20 ng/mL, microglia were monitored for LDH excretion over time (4, 24, 48, and 72 h), 250,000 cells/well, 12‐well plate. CyQuant LDH Cytotoxicity assay (Invitrogen, C20300) was performed, using both positive control and maximum LDH release. The samples were analyzed with a Wallac Victor2 1420 Multilabel Counter (Perkin Elmer). Cytotoxicity % calculated: (sample‐media/max LDH‐media) ×100%. The concentration of 0.5 μM was selected utilizing previous studies together with these experiments, to be the lowest, nontoxic dose, that induced noticeable functional changes (Appendix S1, Figure 1d and e).

### 4.5 RT‐qPCR

Microglia were plated 500,000 cells/3.5 cm dish (Matrigel coated). For characterization the cells were untreated. To assess the exposure effect, 24 h IFNγ 20 ng/mL pre‐treatment, followed by 4 h αSyn PFFs 0.5 μM was used. RNA was collected over ice, cells rinsed once with cold PBS. Lysis buffer was added over the cells, and they were scraped to Eppendorf tubes in RT. RNA extraction was performed directly after with RNeasy MiniKit (Qiagen, 74,104). The RNA concentration was measured with NanoDrop DS‐11 FX (Denovix) and converted to cDNA with Maxima Reverse Transcriptase (Thermo Scientific, EP0741). To quantify the relative expression of genes of interest Maxima probe/ROX qPCR master mix (Thermo Scientific, K0233) with TaqMan primers (Thermo Fisher Scientific, GAPDH HS99999905_m, P2RY12 Hs00375457_m1, PELI1 Hs00221035_m1, TLR4 Hs00152939_m1, TREM2 Hs00219132_m1, AIF1 HS0061049_g1, TMEM119 HS01938723_u1) were used on Step One Plus Real‐Time PCR System (Applied Biosciences). The hiPSC and HPC RNA were extracted the same way. The Ct mean value was normalized to internal Ct mean value GAPDH, and the relative expression presented as a fold change compared to hiPSCs or control microglia. P2RY12 expression was compared to HPCs, as hiPSCs expression was below detection level.

### 4.6 Bulk RNAseq

Exposure time was determined using RT‐qPCR detection of known initiators of phagocytosis TLR2 and TLR4 (4, 6, and 10 h) (Appendix S1, Figure 1b) and cross referencing the results with the functional phagocytosis assay (Appendix S1, Figure 1c, see *Phagocytosis of 465 ATTO‐labeled αSyn PFFs*). At time point 4 h, gene expression of TLRs was at the highest and at this timepoint the phagocytosis process was well on the way. Differentiated microglia cells were plated on Matrigel‐coated 6‐well plate 500,000 cells/well, and matured for 3 days, as described above. Then assay media was changed to the cells with half of the microglia were exposed to IFNγ (20 ng/mL) for 24 h Then the cells were exposed to human αSYN PFF 0.5 μM for an additional 4 h. Cells were lysed over ice (Buffer RLT + β‐mercaptoethanol 10 μL/mL), and RNA was extracted directly after lysis with the Qiagen Rneasy Mini Kit (Qiagen, 74,106) according to the manufacturer's protocol. The samples were further purified and concentrated with RNA Clean & Concentrate (Zymo Research, R1013) including DNAse treatment. The final RNA was eluted with 15 μL DNAse/RNAse‐free water. 5 μL of the sample was put aside for quality control. Quality control was performed combining multiple tools. A spectrophotometer was used to check the RNA purity (260/280 nm >2.0) (DeNovix NanoDrop). The desired minimum RNA amount (600 ng) was measured by fluorometry (Qubit). The RNA integrity was inspected by gel electrophoresis on the 2100 Bioanalyzer (Agilent Technologies) (RIN >7.0) using Agilent RNA 6000 Nano Chip. Only samples that passed all the quality control checks were selected for library preparation.

First, the ribosomal RNA was removed using the RiboCop rRNA Depletion Kit (Lexogen, 144.24). Next, the CorAll Total RNA‐seq Library Prep Kit (Lexogen, K09596) was used to prepare dual indexing for the samples. Quality control was performed using fluorometry, the Qubit dsDNA HS Assay Kit (Qubit, Q32854), Bioanalyzer gel electrophoresis, and High Sensitivity DNA Kit (Agilent, 067–4626). The library was then sequenced in the Genome Center of Eastern Finland (Institute of Clinical Medicine, School of Medicine, Faculty of Health Sciences, Kuopio, Finland), using Illumina NextSeq 500 (NB501525), together with NSQ 500/550 Hi Output KT v 2.5 (75 cys) (Illumina, 20,024,906) and NextSeq Phix Control Kit (Illumina, 15,051,973/FC‐100‐3002).

### 4.7 RNA‐seq data analysis

Bulk RNA sequences of mRNA and miRNA were aligned and quantified to the human genome of reference GRCh38 using the nf‐core workflow (Ewels et al., 2020). Count data was prepared following the workflow defined by Law et al. (Law et al., 2018). The molecules with the lowest expression were filtered out in any condition using the function “filterByExpr” to increase the reliability of the mean–variance relationship. The differences between samples due to the sequencing depth were removed by normalizing the count using the trimmed mean of M‐values (TMM) method (Robinson & Oshlack, 2010) and by applying a log transformation minimizing the sum of sample‐specific squared differences to enhance the true positive and negative ratio in the downstream analysis (Zhang et al., 2019). Any outliers and sample's features inducing batch effect were checked by performing a principal component analysis and unsupervised consensus clustering with Cola (Gu et al., 2021). The quality control revealed that sample preparation was influencing the grouping of samples in the dataset. The batch effect was corrected by using the negative binomial regression from Combat (Zhang et al., 2020). The design matrix for each pair of conditions was created to compare (contrast) and performed the differential expression analysis using limma/edgeR model (Law et al., 2018) controlling for the false discovery rate with the Benjamini‐Hochberg procedure (Benjamini & Hochberg, 1995). From the sequencing data, pairwise comparisons were performed, and DEGs were discovered (Appendix S2). The DEGs of each contrast were uploaded to QIAGEN IPA (QIAGEN Inc., https://digitalinsights.qiagen.com/IPA) (Krämer et al., 2014) for IPA and functional enrichment analysis. The analysis was performed with default parameters, and the IPA background was composed of non‐significant genes. The bubble blots were generated utilizing SRplot (Tang et al., 2023). Lysosomal DEGs were detected by comparing them to the human lysosome database (http://lysosome.unipg.it/).

### 4.8 Phagocytosis of pHRodo labeled bioparticles

Microglia were plated onto Matrigel‐coated 96‐well plates, 20,000 cells/well. The cells were matured for 3 days. 1 mg of the pHRodo zymosan A BioParticles conjugate (Invitrogen, P35364) were resuspended in 2 mL OptiMem (Gibco, 31,985,070) solution and sonicated for 5 cycles (30s ON/30 s OFF) (Diogenode Bioruptor NextGen). Baseline images without the particles were taken first, then pHRodo 5 μg/well added to the wells, and the imaging schedule commenced on IncuCyte S3/SX3 live‐cell‐imaging (Sartorius). Images were taken every 20 min for 6 h.

### 4.9 Preparation of preformed alpha‐synuclein fibrils

The human αSyn PFFs 5 mg/mL were a kind gift from the lab of Kelvin C. Luk. The PFFs were prepared as previously described (Volpicelli‐Daley et al., 2014). The stock was diluted with PBS to 1 mg/mL, then sonicated (Diogenode Bioruptor NextGen) 10 cycles (30 s ON/30 s OFF), stored at −70°C, and then thawed just before use. The αSyn PFFs were handled in the biosafety cabinet, and 1% SDS was used to wipe the surfaces and pipettes prior to normal disinfection procedures after exposures.

### 4.10 Phagocytosis of 465 ATTO‐labeled αSyn PFFs

The αSyn PFFs were labeled by conjugating them with N‐hydroxysuccinimidyl (NHS)‐ester ATTO 465 (ATTO‐TEC, AD 465). Conjugate was prepared in 2 to 3 dye‐to‐protein ratio, and a mixture incubated for 1 h in RT. Conjugate was purified by centrifugation twice at 15000*g*/10 min. Resuspended in PBS, final concentration: 1 mg/mL. Microglia were plated and matured as described above (phagocytosis of pHrodo labeled bioparticles). To assess the appropriate αSyn PFFs concentration 0.1–1 μM concentrations were tested, and phagocytosis was monitored over 6 h (Appendix S1, Figure 1c). For the phagocytosis assay 0.5 μM αSyn PFFs were selected, with the lowest concentration together with clear phagocytic response. Cells were plated as above, (*n* = 3/exposure), assay media was changed, and half of the samples were exposed to IFNγ 20 ng/mL for 24 h. Then the medium was changed to exposure media, with or without 465 ATTO‐labeled αSyn PFFs, with or without IFNγ, and the cells were monitored for 72 h in IncuCyte S3/SX3 live‐cell‐imaging (Sartorius), 10× objective. Brightfield and green fluorescence images were taken every 1 h, two images per well. Results were analyzed with IncuCyte S3 2019B software. Top‐Hat segmentation was used in the quantification of green fluorescence from phagocytosed αSyn PFFs. Phagocytosis index was used to describe the phagocytosis state, this was effectively the cellular area with positive fluorescence divided by total cellular area ×100%.

### 4.11 Scratch wound assay

Microglia were plated onto double coated (PDL 0.05 mg/mL & Matrigel) IncuCyte Imagelock 96‐wellplate (Sartorius, BA‐04856) 100,000 cells/well for 3‐day maturation. 24 h prior to assay, media was changed to assay media with or without IFNγ 20 ng/mL. IncuCyte WoundMaker was used to perform the scratch. The scratch was made to the confluent dish plate, media was changed, and assay medium applied. Assay medium contained: medium alone, αSyn PFFs 0.5 μM, IFNγ 20 ng/mL, αSyn PFFs 0.5 μM + IFNγ 20 ng/mL, or ATP 100 nM. Images were taken in the IncuCyte S3/SX3 live‐cell‐imaging (Sartorius), with 10× objective. One image taken hourly per well for 24 h. Integrated Cell Migration analysis module (Sartorius, 9600–0012) was used to analyze the wound confluency % over time. The migration speed was calculated using 6 and 24 h timepoints (Supplement); Δ wound confluency %/ Δ time.

### 4.12 xCELLigence cellular impedance measurement

Bioimpedance measurement utilizing the xCELLigence Real‐Time Cell Analysis (Agilent Technologies) was used to determine the kinetics of intracellular microglial response to the αSyn PFFs and IFNγ exposures. E‐plates 16 (Acea, 720,061) were coated with Matrigel and after polymerization medium changed to microglia medium. The first baseline for cell index (a relative impedance value derived by the xCELLigence software) was measured without cells. Then cells (20,000 cells/100 μL/well) were plated for 3‐day maturation. Then the medium was changed to exposure media, and the cells were monitored for 48 h, with measurements every 10–30 min. Groups: control, αSyn PFFs 0.5 μM, IFNγ 20 ng/mL, and αSyn PFFs 0.5 μM + IFNγ 20 ng/mL. No group had any pre‐stimulation, the exposures were administered simultaneously. The cell Index was normalized to 1 from the point of exposure (Appendix S1, Figure 1f and g).

### 4.13 Western blot

The microglia were plated onto 24‐well plates with Matrigel‐coating 300,000 cells/well. The exposure groups were control 48 h, αSyn PFFs 0.5 μM 24 h, IFNγ 20 ng/mL 48 h, αSyn PFFs 24 h + IFNγ 48 h. The cells were rinsed 1× ice cold PBS and then collected over ice, by scraping into 50 μL 1× RIPA buffer with Complete EDTA‐free (Roche, 11,873,580,001) and PhosStop (Roche, 04906837001) and 50 μL 2× Laemmli buffer. The samples were then vortexed, boiled for 6 min, cooled on ice, and stored in −20°C until running on gel.

The samples were run on 7.5%–15% SDS‐Page gel (5 μL/well). The immunoblotting was performed utilizing Transblot Turbo Transfer System according to the manufacturer's instructions, together with TransBlot Turbo Transfer Pack Midi format, 0.2 μM PVDF (Biorad, 1,704,157). The membranes were blocked with skimmed milk in 1×TBS/0.1%tween (TBST) 30 min in RT. Primary antibodies were introduced in 50 mL tubes with the membranes inside and incubated on rollers overnight at 4°C. Secondary antibodies were incubated on bellydancer for 2 h at RT. Detection of the bands was performed using ECL Plus Western Blotting Substrate (Pierce, 32,134), Supersignal West Pico Plus Chemiluminescent Substrate (Thermo Scientific, 34,577) or Cy‐fluorophore and Biorad ChemDoc MP imaging system.

The primary antibodies were in 5% BSA in TBST 1:1000 and the secondary antibodies in blocking buffer 1:5000 (HRP) or 1:2000 (Cy3/Cy5). The primary antibodies used were anti‐TOM20 (Cell Signaling, D8TYN), anti‐αSyn (BD Transduction laboratories, 610,787), anti‐Cytochrome C (BD Pharmingen, cat. 5,565,433), anti‐β‐actin (Sigma, A5441), anti‐iNOS (Invitrogen, MA5‐17139), anti‐TRL4 (Novus Biologicals, NB100‐56580), anti‐TREM2 (R&D, AF1828), anti‐LAMP1/CD107a antibody (Novus Biologicals, NBP2‐25154SS), anti‐LAMP2/CD107b (Novusbio, NB300‐591), anti‐Beclin1 (Novus Biologicals, NB500‐249), anti‐p62 (Cell Signaling Technology, 5114S), anti‐MnSOD (Assay Designs, ADI‐SOD‐110), and 1:3000 anti‐LC3B (Abcam, Ab51520), and the secondary antibodies anti‐rabbit IgG HRP‐linked antibody (Cell Signaling, 04/2016), anti‐mouse HRP‐linked antibody (Sigma, A9044), anti‐mouse Cy3 (Sigma, PA43009V), anti‐rabbit Cy5 (Sigma, PA45011V).

### 4.14 Cytokine detection

Cells were plated on Matrigel coated 6‐well plates, 700,000 cells/well. Medium was collected 96 h post‐exposure: control, αSyn PFFs 0.5 μM 72 h, IFNγ 20 ng/mL 96 h, αSyn PFFs 0.5 μM + IFNγ 20 ng/mL 72 h + 96 h, matching the end of phagocytosis assay (see *Phagocytosis of 465 ATTO‐labeled αSyn PFFs*). The Human Cytokine Bead Array Kit (BD, 558264) was used to detect the secreted cytokine levels in the media samples. Medium samples were collected over ice, centrifuged, and directly frozen to −70°C. The samples and cytokine standards were incubated with 1:75 bead mixture. Capture beads utilized: Human IL‐1β (BD, 558279), Human IL‐8 (BD, 558277), Human MIP‐1α (BD, 55827), Human MCP‐1 (BD, 55827), Human IL‐6 (BD, 560112), Human IL‐10 (BD, 558274), then incubated with 1:75 detection reagent. CytoflexS (Becman Coultier) flow cytometer was used to run the assay, minimum of 350 beads captured/cytokine. The cell populations were detected using APC‐A with APC‐A750‐A. PE‐channel was used to detect the levels of individual populations. FCAP Array v 2.0.2 (SoftFlow) was used to analyze the cytokine concentrations comparing to the known standards. Synergy was calculated as follows: (([αSyn PFFs] + [IFNγ])‐control) < (αSyn PFFs + IFNγ).

### 4.15 Glucose uptake assay

Microglia were plated onto Matrigel‐coated 96‐well plate 100,000 cells/well. Matured as above. Cells were pre‐stimulated with IFNγ (20 ng/mL) for 24 h then 0.5 μM αSyn PFFs were introduced for additional 24 h. Cells then were detached with Accutase (StemCell Technologies, 07920), and collected to 96‐well round bottom plate (Corning Incorporated, 3799) by rinsing with MG‐medium. Glucose Uptake Cell‐Based Assay Kit (Cayman Chemicals, 600,470) was used to detect glucose uptake. Centrifugation 400*g*/5 min RT was used to separate the media/treatments from the cells. Supernatant removed and media changed to glucose free DMEM (Gibco, 11,966,025) and incubated for 20 min at 37°C. 200 μg/mL 2‐NBDG (2‐(N‐(7‐Nitrobenz‐2‐oxa‐1,3‐diazol‐4‐yl)Amino)‐2‐Deoxyglucose) fluorescently labeled glucose derivative was added to the wells and incubated further 10 min at 37°C. Propidium Iodide (PI, 1:1000) was used to detect the dead cells. CytoflexS (Becman Coultier) was used to detect the fluorescence; FITC (2‐NBDG) and EDC (Propidium Iodide). Cell controls for no stain, only PI and only 2‐NBDG were used. Dead cells were excluded by cell count to EDC fluorescence; live cells did not stain (Appendix S1, Figure 7c). The 2‐NBDG positive cells were gated using FITC‐A with SSC‐A (Appendix S1, Figure 7d). The 2‐NBDG fluorescence mean was divided by the events detected. Results given as Log2 fold change from the controls.

### 4.16 Data quantification and statistical analysis

GraphPad Prism 9.0.0 (121) (GraphPad Prism Software, LLC) was used to analyze the statistical significance. One‐way‐ANOVA was used. Post hoc testing used was Tukey's multiple comparisons. Levels of significance *p < 0.05; **p < 0.01; ***p < 0.001 were used. SD was used to describe the error within samples. Grubbs test was utilized to identify significant outliers.

## AUTHOR CONTRIBUTIONS

Jonna Niskanen, Šárka Lehtonen, Gundars Goldsteins, and Katrina Albert conceptualized the study. Jonna Niskanen performed experiments and analyzed data with assistance from the following: Sanni Peltonen assisted in culturing, harvesting, and differentiating the cells and collecting samples. Sohvi Ohtonen assisted in IncuCyte studies. Mohammad Feroze Fazaludeen performed RNAseq library preparation and Luca Giudice. RNAseq analysis. Kelvin C. Luk provided the αSyn PFFs, Jari Koistinaho provided material, Tarja Malm provided resources. Jonna Niskanen, Katrina Albert, Šárka Lehtonen drafted the manuscript. Gundars Goldsteins provided critical input to the draft manuscript. All authors read and approved the final version of the manuscript.

## Supporting information


**Appendix S1:** Supporting information.

**Appendix S2:** Supporting information.

**Data S1:** Supporting information.

## Data Availability

The data that support the findings of this study are openly available in GEO at https://www.ncbi.nlm.nih.gov/geo/, reference number GSE277636.
