# Supplementary material for: Uptake of alpha‐synuclein preformed fibrils is suppressed by inflammation and induces an aberrant phenotype in human microglia
Source: Glia. 2024 Oct 22;73(1):159–74. doi: 10.1002/glia.24626 (PMC11660540; doi:10.1002/glia.24626)
Supplement: Supplementary file 1 — Appendix S1: Supporting information. [file GLIA-73-159-s001.pdf]

Appendix S1

Supplementary figures and images

Table S 1 hiPSC lines utilized in this study

Line	Gender	Age at biopsy	Reference
Ctrl3	F	44	Oksanen et al. 2017
Mad 6	M	63	Fagerlund et al. 2022
ChiPSC7	F	20	Takara Bio, Y00275
ChiPSC22	M	32	Takara Bio, Y00325

Table S 2 hiPSC-lines used in each experiment

Line	qPCR	ICC	CBA	Phagocytosis	Scratch wound	RNAseq	LDH	WB	xCelligence	Glucose uptake
Ctrl3	x	x	x	x	x	x		x	x	x
Mad 6	x	x		x		x	x			
ChiPSC7	x	x								
ChiPSC22	x	x					x		x	x

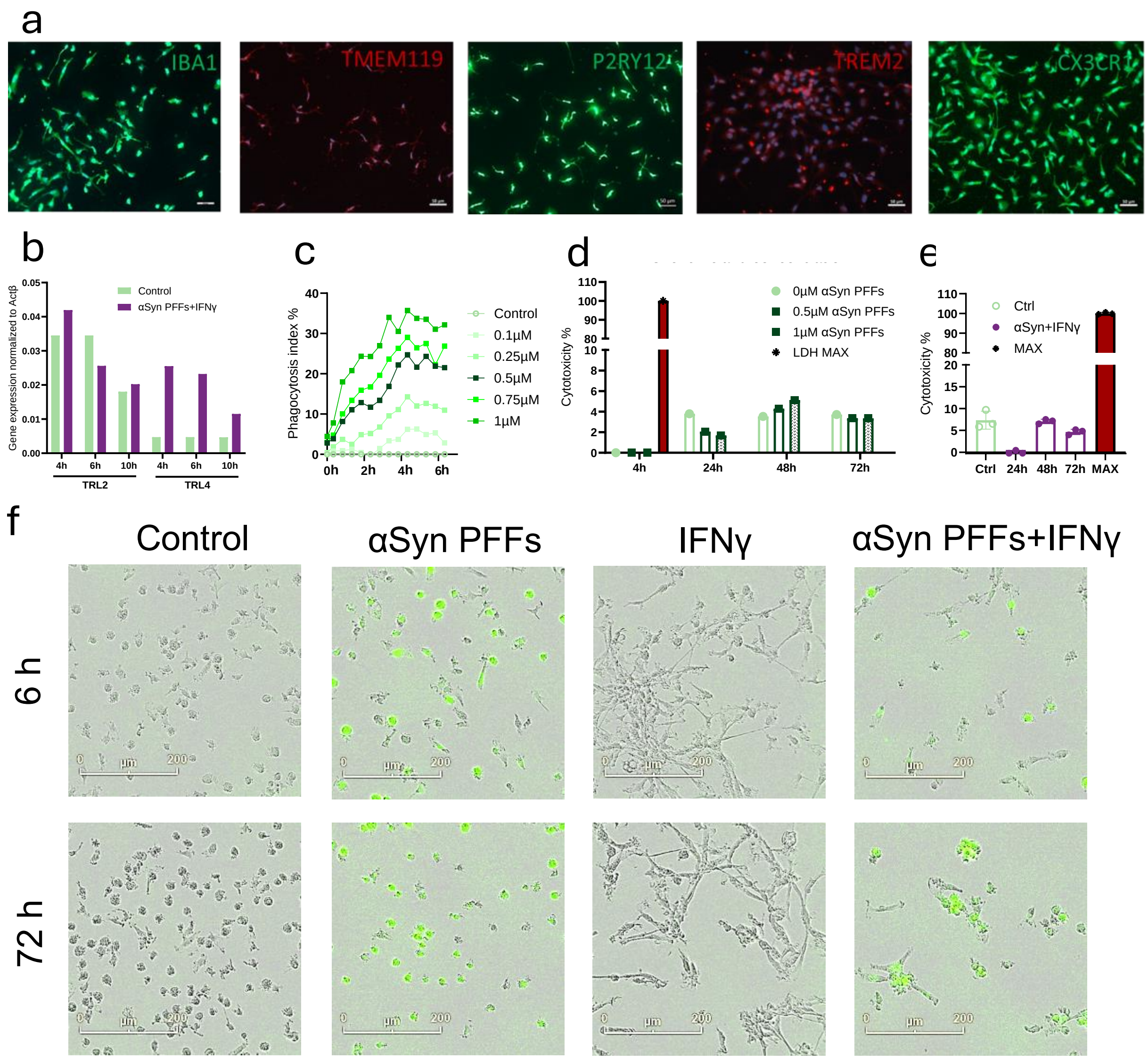

Figure S 1 (a) *Representative ICC images of microglial markers IBA1 (green), TMEM119 (red), P2RY12 (green), TREM2 (red), and CX3CL1 (green). Scale bar 50µm.* ***(b)*** *Gene expression of TLRs over time.* ***(c)*** *Phagocytosis of ATTO 465-labeled αSyn PFFs with different concentrations.* ***(d)*** *LDH release measurement used as indicator for cytotoxicity. 0.5µM and 1µM concentrations tested over time.* ***(e)*** *LDH release used to detect cytotoxicity of 0.5µM αSyn PFFs + IFNγ 20ng/ml.* ***(f)*** *Microglia morphology from phagocytosis assay at 6 h and 72 h post ATTO 465 -labeled 0.5µM αSyn PFFs exposure. The cells were pretreated with 20ng/ml IFNγ 24 h before the start of imaging. Scalebar 400 µm.*

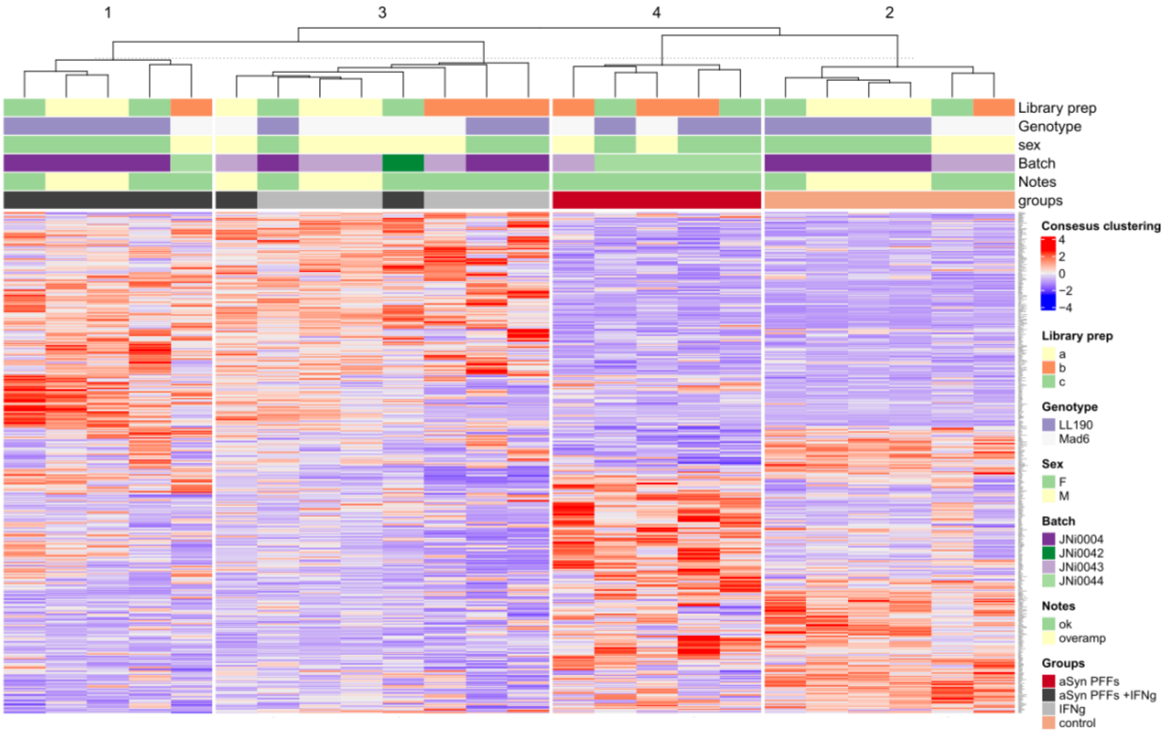

Figure S 2 *Consensus clustering from the transcriptomics data indicating similarities between the samples considering batch, individual (genotype, sex), and exposure variables. Cells were collected from four differentiation batches (JNi0004, JNi0042, JNi0043, JNi0044). Two genotypes were used, a male and a female. The sequencing library was created in 3 batches. Exposure groups were control, 0.5 µM αSyn PFFs 4 h, IFNγ 20 ng/ml 28 h, and 0.5 µM αSyn PFFs 4 h with IFNγ 20 ng/ml 28 h. The inflammatory stimulus (IFNγ) clearly activated the microglia, whereas αSyn PFFs alone was clustering closer to control microglia.*

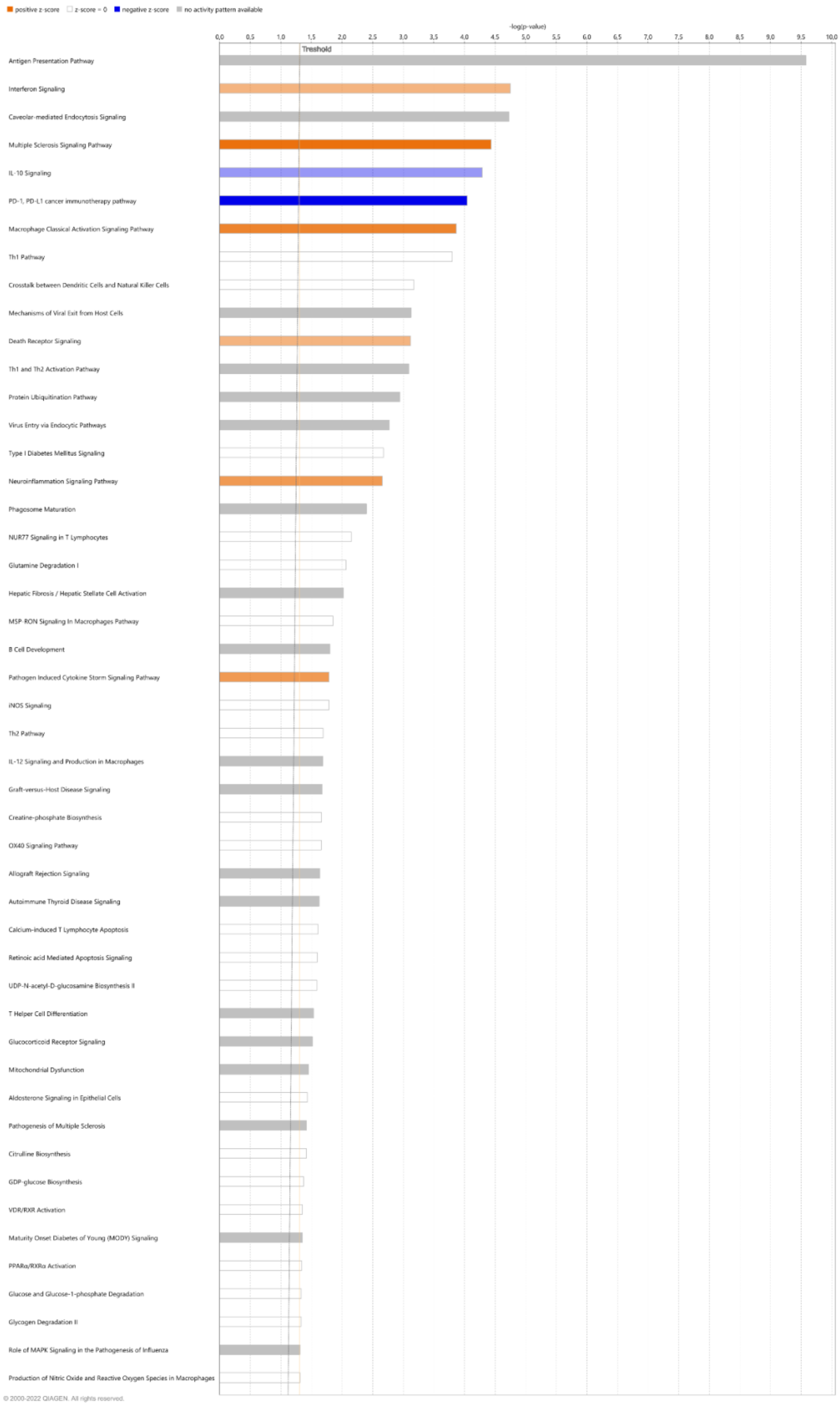

Figure S 3 *IPA canonical pathway analysis from RNAseq data displaying comparison group αSyn PFFs + IFNγ vs. αSyn PFFs*

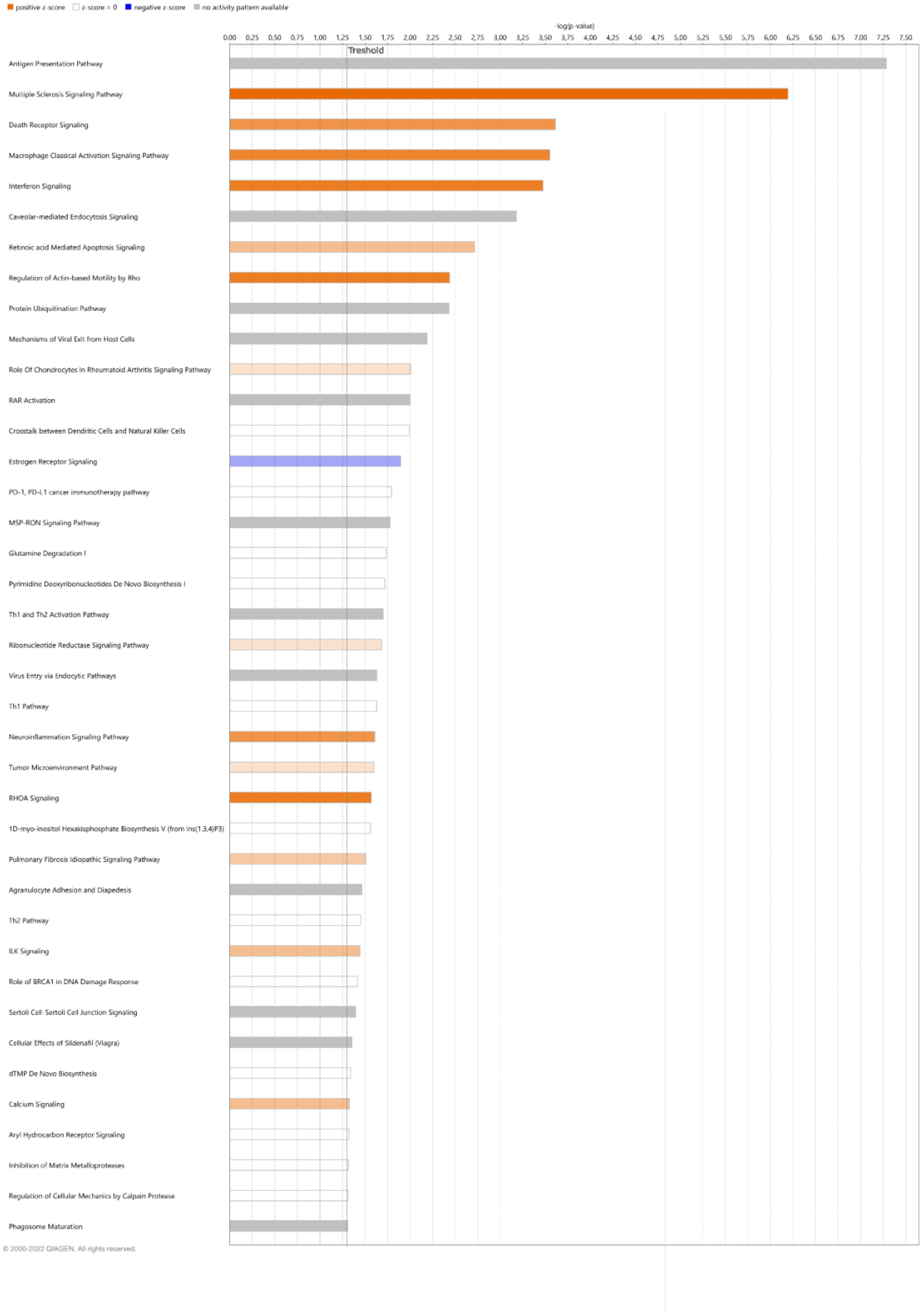

Figure S 4 *IPA canonical pathway analysis from RNAseq data displaying comparison group αSyn PFFs + IFNγ vs. control*

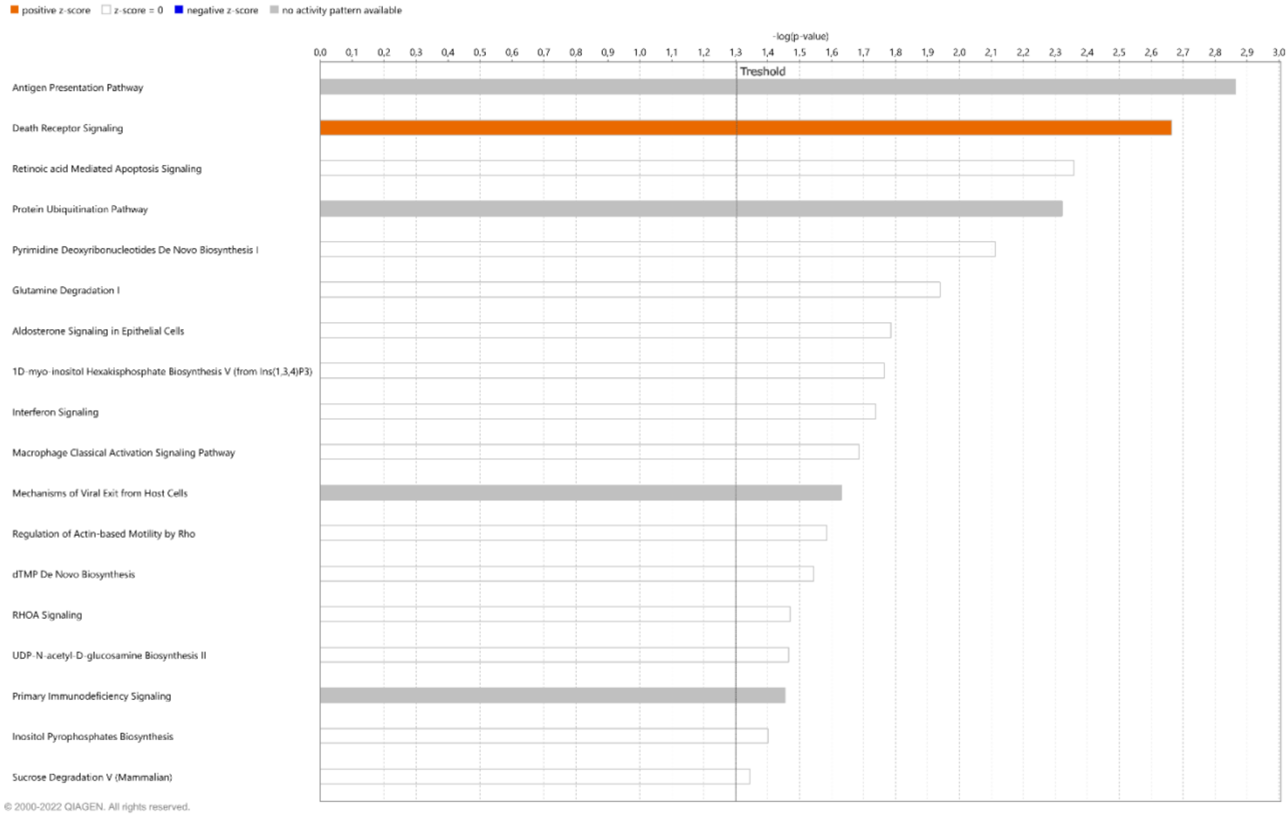

Figure S 5 *IPA canonical pathway analysis from RNAseq data displaying comparison group IFNγ vs. control.*

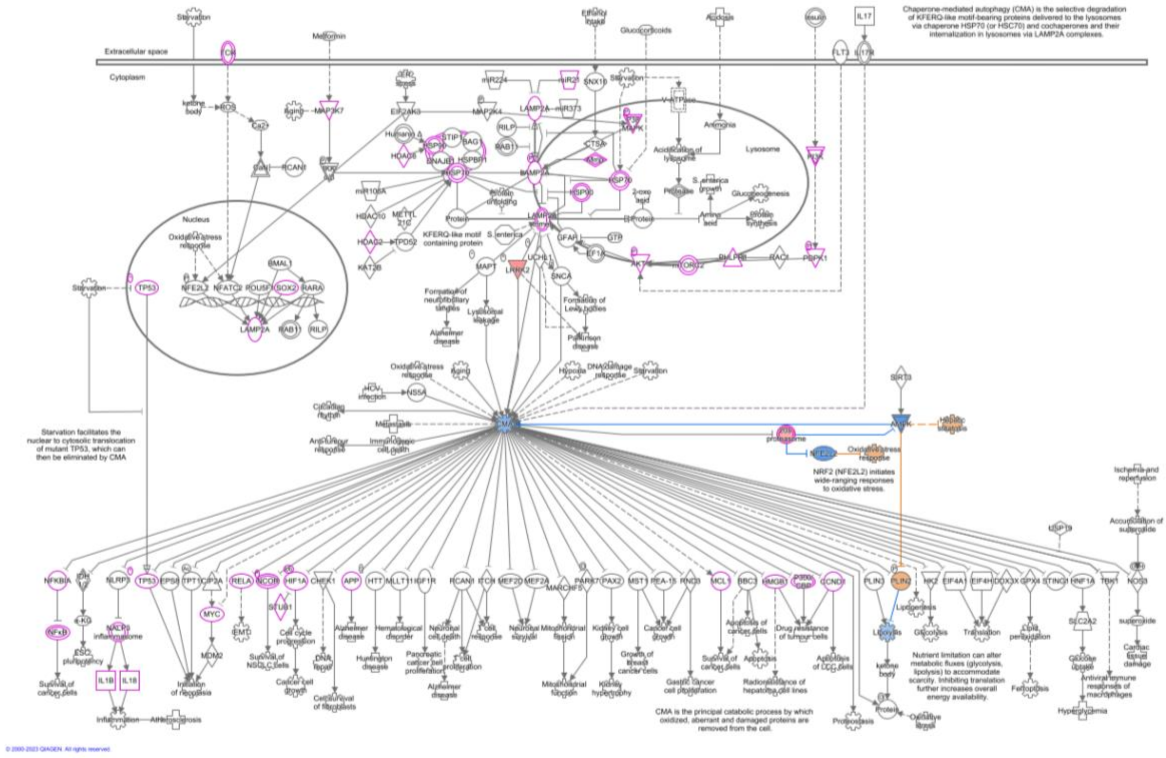

Figure S 6 *IPA αSyn degradation pathway from the groups αSyn PFFs+ IFNγ compared to αSyn PFFs.*

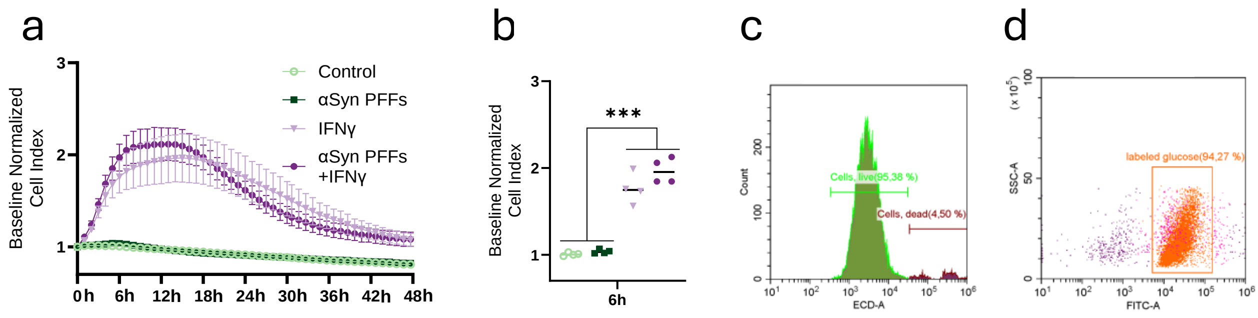

Figure S 7 (a) *xCELLigence measurement to assess the cellular impedance. All stimulants given at timepoint 0h.* ***(b)*** *Statistically significant changes in the microglial cellular impedance detected at 6h timepoint with xCELLigence. Error bar indicating SD. One-way-ANOVA with Tukey's multiple comparisons was used to determine statistical significance. p<0.05=* p<0.01=**, p<0.001=***.* ***(c)*** *Propidium Iodide (ECD-fluorescence) was used to detect the live cells.* ***(d)*** *The 2-NBDG positive cells (FITC-fluorescence) were gated using SSC-A and FITC-A.*
