# Supplementary material for: Uptake of alpha‐synuclein preformed fibrils is suppressed by inflammation and induces an aberrant phenotype in human microglia
Source: Glia. 2024 Oct 22;73(1):159–74. doi: 10.1002/glia.24626 (PMC11660540; doi:10.1002/glia.24626)

Supplementary file for the full uncropped Western Blots

The samples are labeled as follow:

Controls = 1, 2, 3
αSyn PFFs = 4, 5, 6
IFNγ = 7, 8, 9
αSyn PFFs+IFNγ = 10, 11, 12
αSyn control sample with only fibrils=PFF

Multiple stainings were done on the same blot with different antibodies in different hosts (e.g., anti-rabbit, anti-mouse) without stripping the membrane between the stainings. The order of the images indicates the order in which they were stained.

Figure 3e: TREM2 and βactin (note: the upper bands from Beclin1 staining – see below Figure 4e)

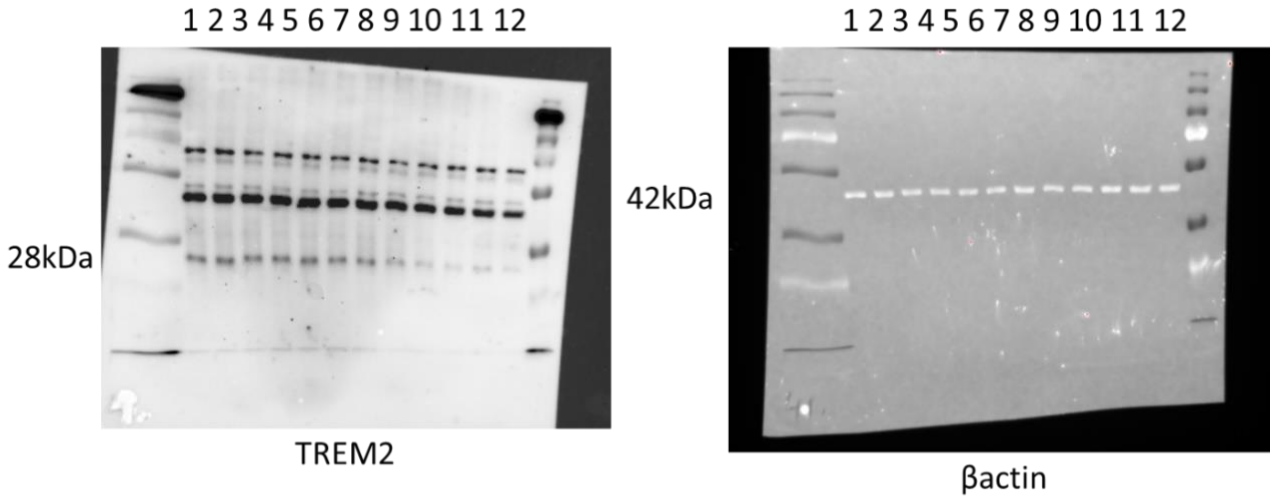

Figure 3f: TLR4 and βactin

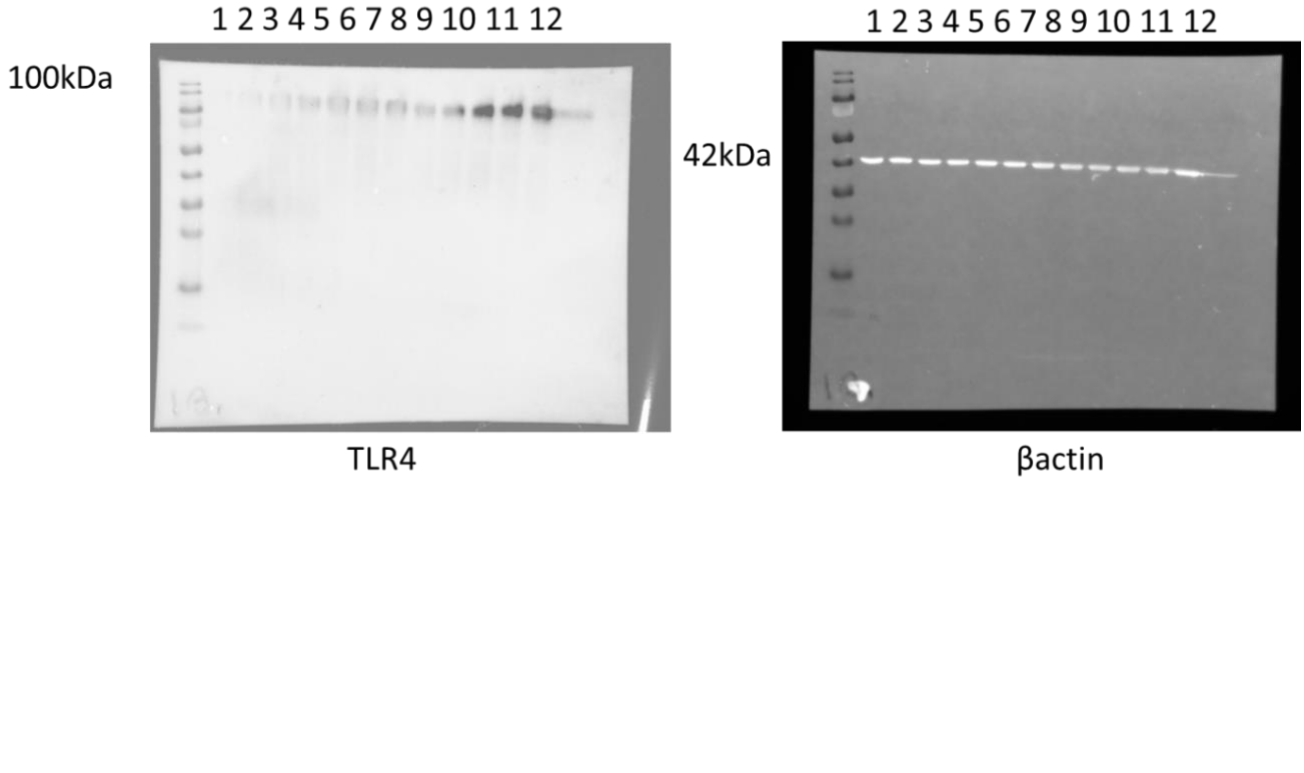

Figure 4b: LC3B and βactin

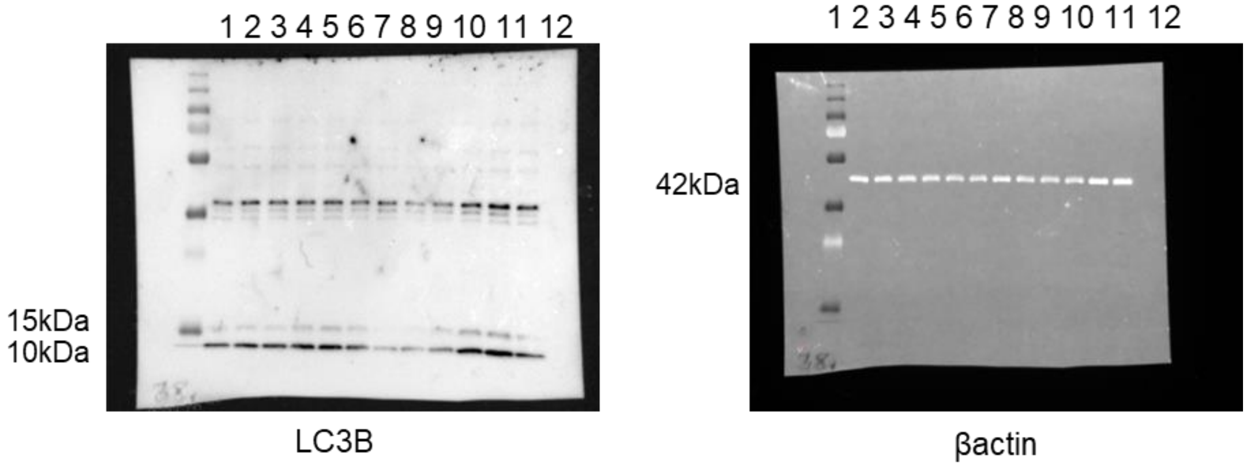

Figure 4d: p62 and βactin

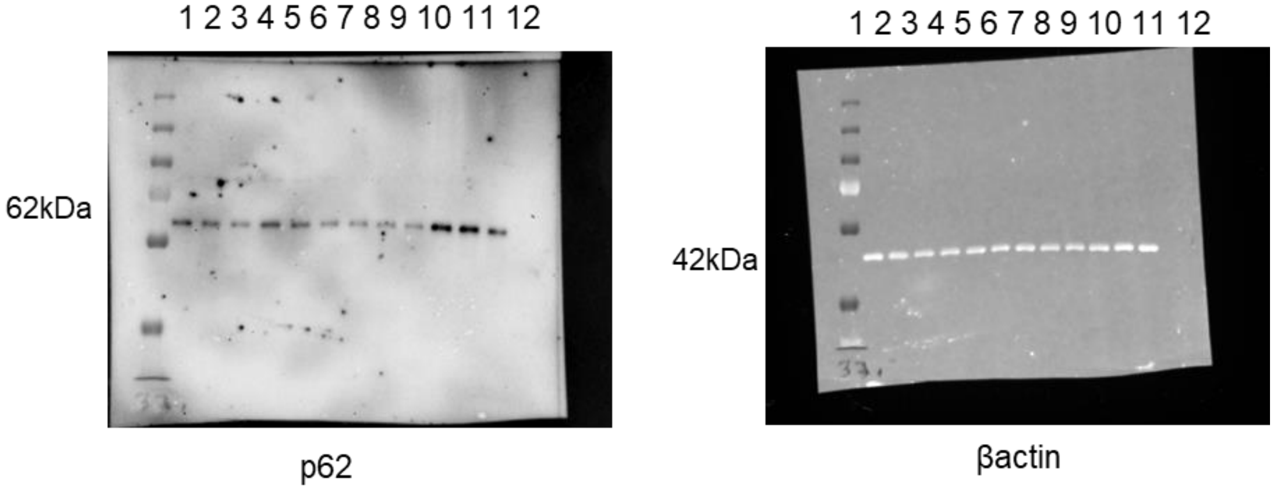

Figure 4e: Beclin1 and βactin

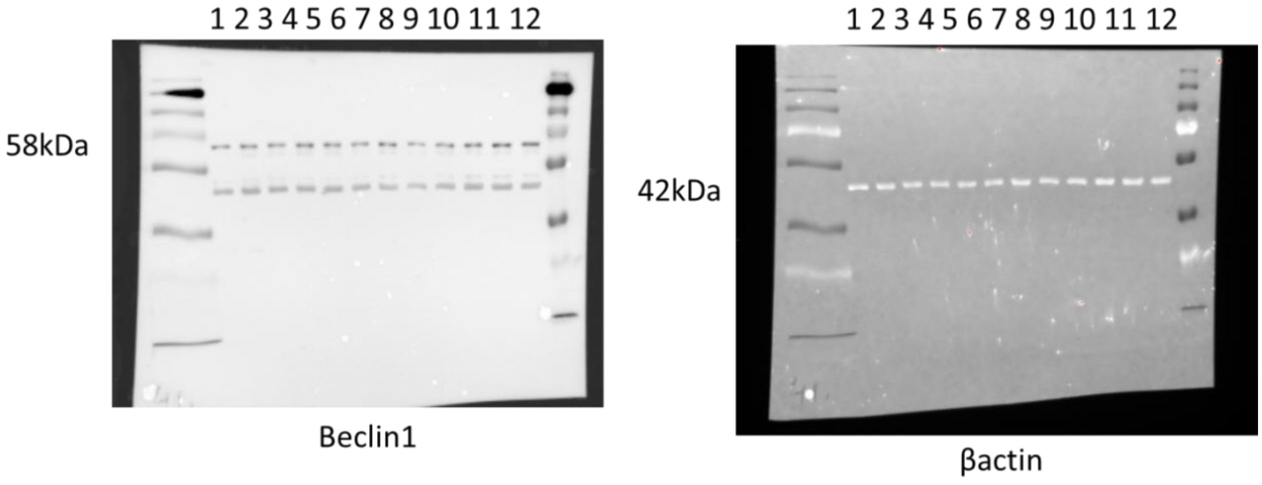

Figure 4g: Total αSyn and βactin

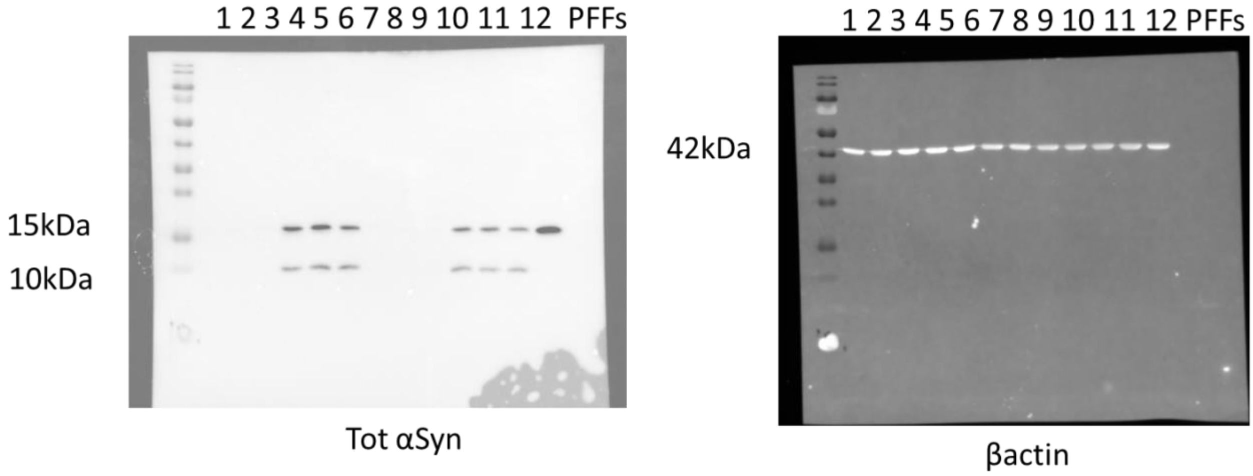

Figure 4i: LAMP1 and βactin

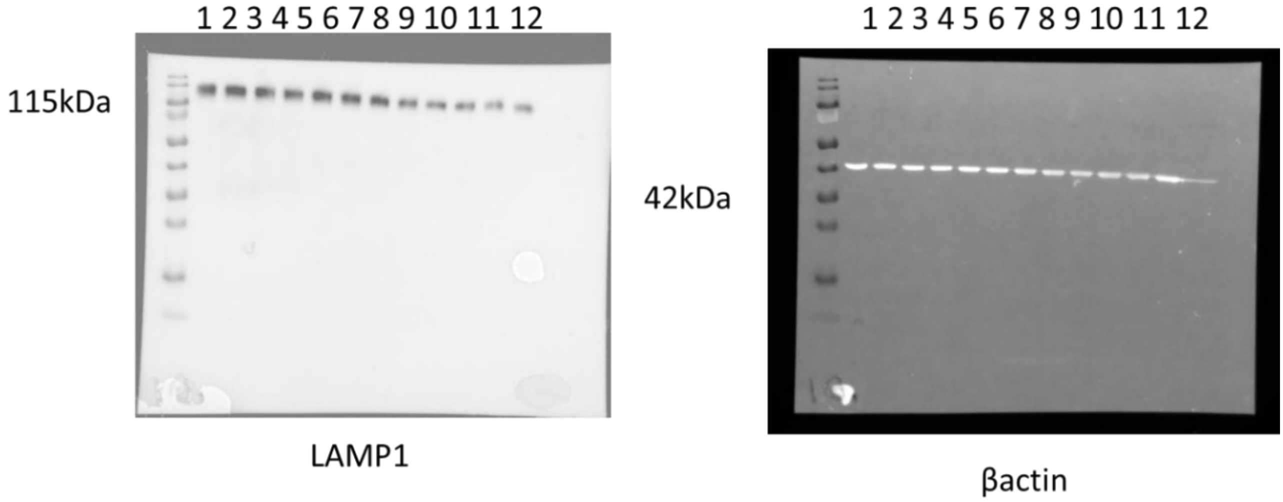

Figure 4j: LAMP2 and βactin (NOTE: sample 1 pipetted twice due to small leakage into ladder well)

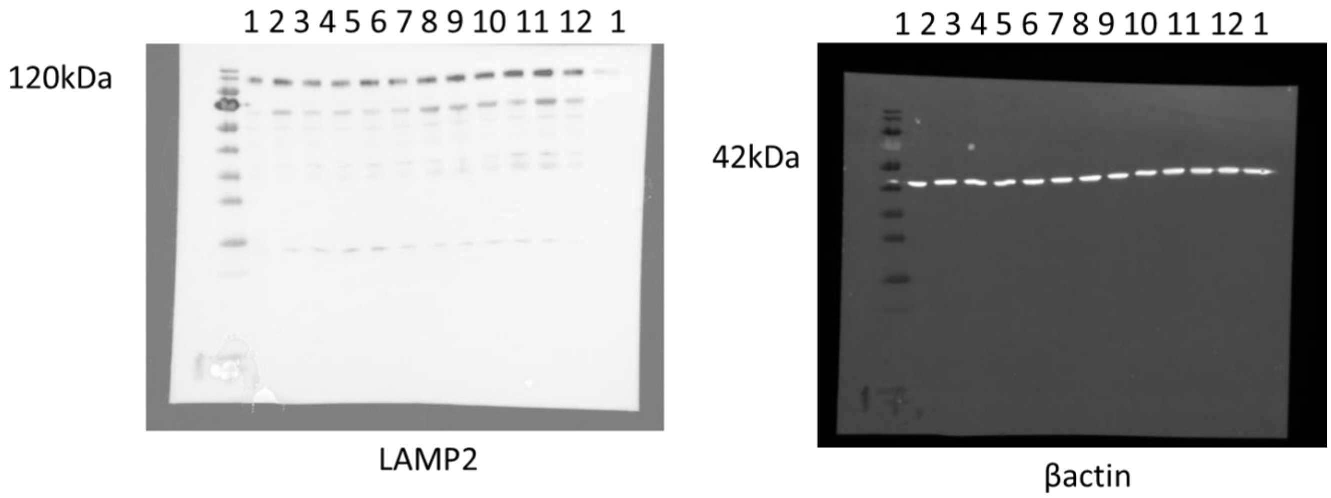

Figure 5b: Cytochrome C, TOM20, and βactin

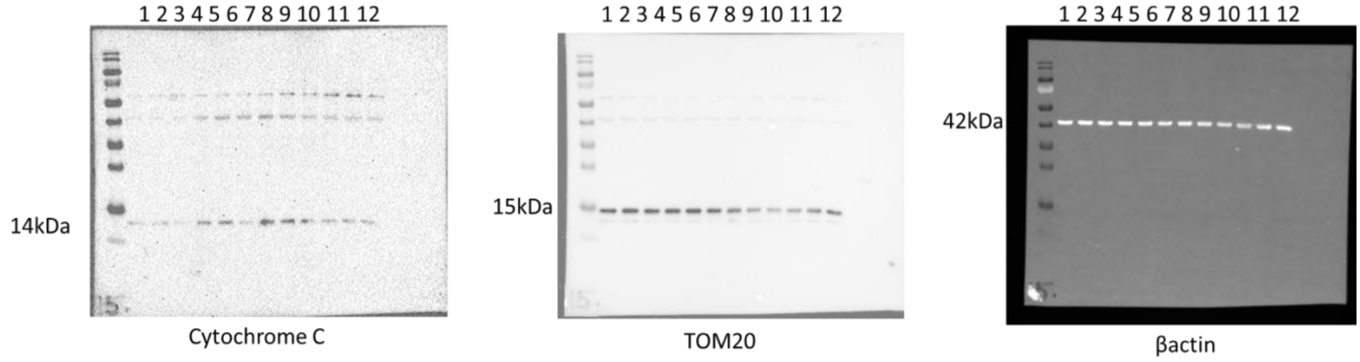

Figure 5c: iNOS and βactin

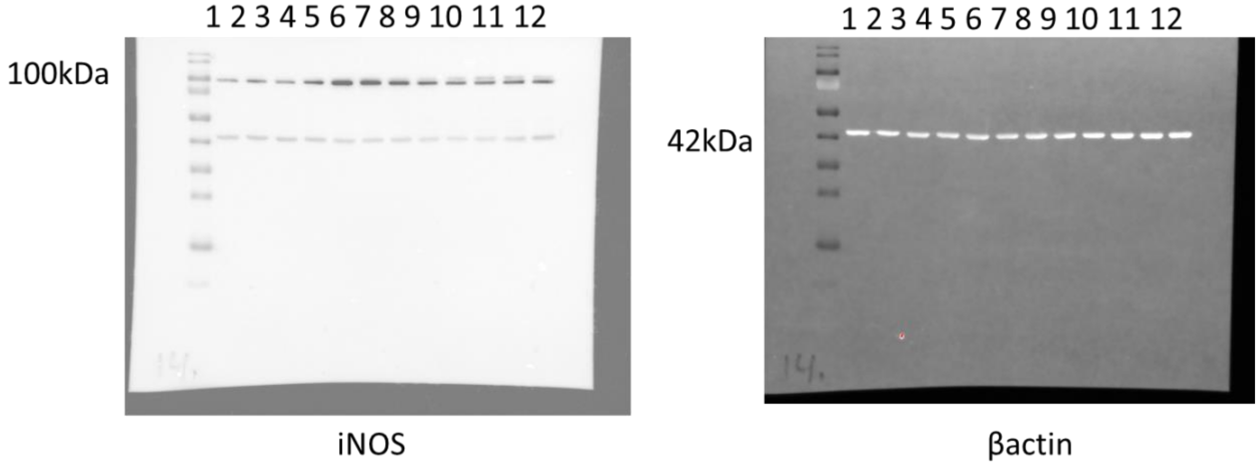

Figure 5d: SOD2 and βactin

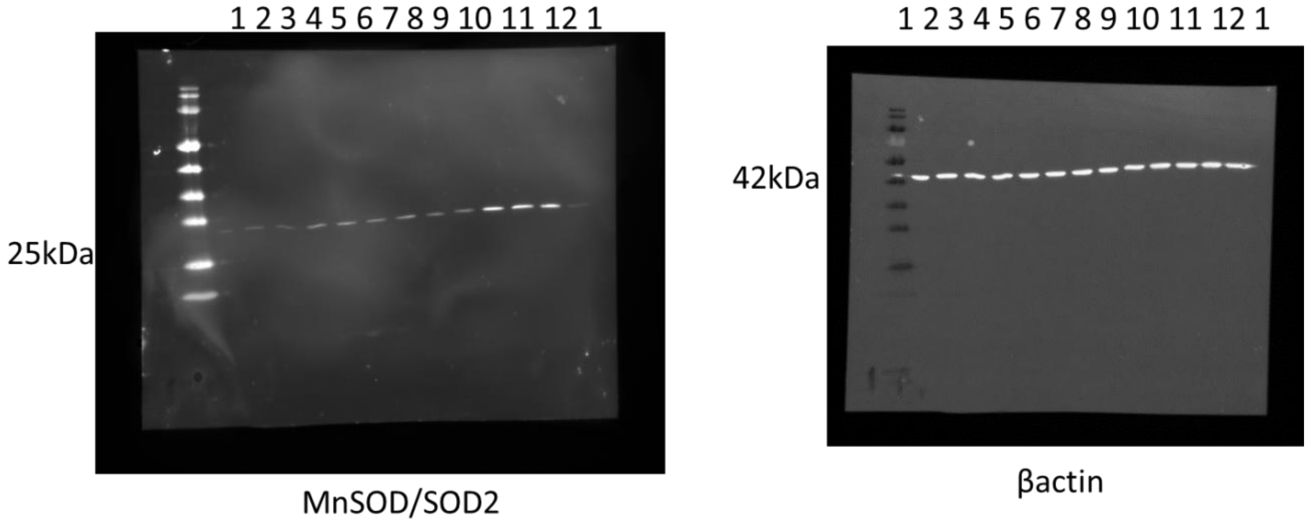

Supplement: Supplementary file 3 — Data S1: Supporting information. [file GLIA-73-159-s002.pdf]
